# Characterization of Key Odorants During Processing of Minty-like Aroma ‘Rucheng Baimaocha’ Black Tea

**DOI:** 10.3390/foods14111941

**Published:** 2025-05-29

**Authors:** Jian Ouyang, Ronggang Jiang, Qi Liu, Hongyu Chen, Xiaoqin Yi, Yuzi Yang, Fangfang Huang, Juan Li, Haitao Wen, Ligui Xiong, Jianan Huang, Zhonghua Liu

**Affiliations:** 1 Key Laboratory of Tea Science of Ministry of Education, Hunan Agricultural University, Changsha 410128, China; jouyang2022@163.com (J.O.); jiangronggang1997@126.com (R.J.); is.liuqi@outlook.com (Q.L.); chenhongyu9805@163.com (H.C.); xiaoqinyi1101@foxmail.com (X.Y.); 15344482128@163.com (Y.Y.); sync@stu.hunau.edu.cn (F.H.); xixi_lj@hunau.edu.cn (J.L.); whtzz731@163.com (H.W.); xiongligui@hunau.edu.cn (L.X.); 2 National Research Center of Engineering Technology for Utilization of Functional Ingredients from Botanicals, Hunan Agricultural University, Changsha 410128, China; 3 Co-Innovation Center of Education Ministry for Utilization of Botanical Functional Ingredients, Hunan Agricultural University, Changsha 410128, China; 4 Key Laboratory for Evaluation and Utilization of Gene Resources of Horticultural Crops, Ministry of Agriculture and Rural Affairs of China, Hunan Agricultural University, Changsha 410128, China

**Keywords:** ‘Rucheng Baimaocha’ black tea, key odorants, processing, GC × GC-O-Q-TOF-MS

## Abstract

The characteristic minty-like aroma of ‘Rucheng Baimaocha’ black tea (RCBT) enhances the tea’s unique flavor profile, driving high demand among consumers. The dynamic changes in key aroma compounds in minty-like RCBT were elucidated by sensory evaluation and gas chromatography olfactometry quadrupole time of flight mass spectrometry (GC × GC-O-Q-TOF-MS). The results indicated that during processing, the aroma of RCBT transitions from a fresh to floral, sweet, and minty-like aroma. Among the 189 identified volatile compounds, alcohols constitute the predominant category (over 50%), with 71 compounds identified as key differential compounds across all stages. Aroma analysis revealed that 28 compounds with odor activity values (OAV) > 1 were the primary contributors during RCBT processing. Notably, minty-like odorants in RCBT were primarily derived from the metabolic pathways of the methylerythritol phosphate (MEP) and mevalonic acid (MVA), lipid oxidation, and phenylalanine. These findings offer theoretical insights for improving unique black tea quality and optimizing processing techniques.

## 1. Introduction

Black tea ranks among the most consumed beverages worldwide, valued for its remarkable health benefits and flavor quality [1,2]. Aroma, as a key indicator of flavor, is indispensable in consumer’s tea selection [3,4]. It not only contributes significantly to the sensory experience but also reflects the product’s market competitiveness. Moreover, specialty variety black teas are particularly favored for their harmonious aromas and flavors [5,6]. The ‘Rucheng Baimaocha’ (*Camellia pubescens*) is a distinctive variety suitable for making both black tea and white tea. The black tea produced from ‘Rucheng Baimaocha’ (RCBT) exhibits floral and sweet notes, accompanied by a distinctive minty-like aroma, setting it apart from other black teas [7,8]. The minty-like aroma endows RCBT with unique flavor characteristics, giving it high recognizability and consumer appeal in the market. Previous studies have shown that 12 key aroma compounds were crucial in shaping the aroma profile of RCBT, as identified through gas chromatography–olfactometry (GC-O), odor activity value (OAV) calculation, and aroma recombination and omission [8]. Of these, four aroma compounds (methyl salicylate, (2*E*,6*Z*)-nona-2,6-dienal, methyl geranate, and (2*E*)-non-2-enal) were identified as crucial contributors to RCBT’s minty-like scent, exhibiting significantly higher concentrations and OAVs compared to those found in other black teas [8]. The minty-like aroma of RCBT has attracted considerable attention, but the dynamics of its key odorants throughout the production phase remain unclear, thus limiting the quality control of RCBT.

The distinctive fragrance of tea originates from volatile compounds that are naturally present in fresh leaves, as well as those produced through biochemical transformations of precursor substances during the manufacturing process [9]. Throughout the entire production process of black tea, the volatile compounds present in fresh leaves undergo notable changes in the content and type [9,10]. Components such as sugars, amino acids, fatty acids, and carotenoids in tea leaves can generate a wide variety of aromatic compounds through complex oxidative degradation and transformation reactions [9,11]. Especially during black tea processing, volatile components (such as alcohols, aldehydes, and esters) undergo significant transformations under the synergistic effects of enzymatic reactions and thermal chemical reactions, resulting in the characteristic floral, fruity, and sweet aromas [10,12,13]. This series of biochemical reactions not only alters the chemical composition of the tea leaves, but also shapes the unique and captivating flavor profile of black tea [14,15,16]. Currently, some studies have explored the volatile or aroma components of RCBT. It has been found that RCBT develops distinct floral and sweet aroma after 4–8 h of fermentation, and extending the process beyond 10 h compromises aroma intensity [17]. As the fermentation time was extended, the relative amounts of nerol, geraniol, methyl geranate, and *β*-ionone in RCBT increased, while the contents of leaf acetate and hexyl caproate decreased [18]. Ouyang et al. [7] investigated the key odorants of ‘Baimaocha’ black tea from different origins. In addition, the relative contents of fruity and floral compounds in black tea processed by ‘Rucheng Baimaocha’ No.1 and No.2 strains were much higher than those in black tea of ‘Zhuyeqi’ variety [19]. However, current research mainly focuses on comparisons of the proportions and contents of volatile compounds in RCBT under different processing techniques or from different varieties, with few studies investigating the changes in key aroma compounds during the processing of RCBT. Recently, the combined analysis of OAV and GC-O has become a powerful way to identify key aroma compounds [5,20]. OAV serves as an essential parameter for assessing the impact of an individual volatile on the overall aromatic profile, while GC-O can combine chromatographic separation with sensory evaluation to detect odor contribution molecules in complex food systems [21].

The primary objective of this investigation was to characterize the critical aroma components of RCBT using gas chromatography olfactometry quadrupole time of flight mass spectrometry (GC × GC-O-QTOFMS). Meanwhile, it attempts to explore the dynamic changes in patterns of aroma during the processing of RCBT with minty-like characteristics. The findings will provide a scientific basis for the quality control and process improvement of RCBT.

## 2. Materials and Methods

### 2.1. Chemicals

The n-alkanes (C7–C25) and ethyl decanoate were purchased from Sigma-Aldrich (St. Louis, MO, USA). Specific details of the aroma standards used in the experiments are the same as in our previous studies [8].

### 2.2. Tea Samples

The one bud and two leaves of ‘Rucheng Baimaocha’ were collected at the end of March 2023 in Rucheng County, Hunan Province. Immediately after the leaves were picked, the black tea was processed at Mucaoren Tea Industry Co., Ltd. (Chenzhou, China). The production process of RCBT began by evenly spreading around 50 kg of freshly picked tea leaves onto specially designed withering trays, maintaining a layer thickness of approximately 2 cm. The leaves underwent a 12 h withering process in a controlled environment (20–25 °C; humidity of 60–68%) until the moisture content reached 58.50%. Subsequently, the withered leaves were uniformly placed into rolling machines (model 6CR-55) for non-pressurized rolling for 25 min. After loosening, the rolled leaves were uniformly layered on circular fermentation trays to a depth of approximately 10 cm and fermented for 6 h. Finally, the fermented leaves were initially dried on a chain dryer and then fully dried in a fragrance-enhancing machine at 80 °C. During the RCBT processing, fresh leaves (FLs); samples after withering for 4 h, 8 h, and 12 h (labeled as W1, W2, and W3, respectively); rolling sample (R); samples after fermentation for 1.5 h, 3 h, 4.5 h, and 6 h (labeled as F1, F2, F3, and F4, respectively); and drying sample (D) were collected. To ensure the representativeness of the samples, three samples were collected at each time point, using a five-point sampling method. The collected samples were immediately frozen in liquid nitrogen for storage and transportation. The non-volatile matrix preparation method in the RCBT processing was the same as in our previous research [8]. Additionally, it was prepared together with the previous ‘volatile-free’ matrix preparation [8].

### 2.3. Sensory Analysis

The sensory assessment of RCBT was performed by 12 experienced reviewers (aged 25–40). Before the formal trial commenced, each evaluation expert underwent specific training using standard specimens and was informed of all the trial details. The sensory analysis was conducted following the GB/T 23776-2018 standard methodology [22]. In brief, 3 g of each tea sample was infused in 150 mL of boiling water for 5 min. Then, the tea infusion was filtered, and the aroma was evaluated by assessors.

### 2.4. Volatile Analysis

The volatiles from all processed tea samples were concentrated by solid-phase micro-extraction (SPME), following the methodology established in our prior research [23]. In brief, a precisely measured amount of 0.5000 g of tea powder was carefully placed into a 20 mL headspace vial. Sequentially, 5 mL of boiling water and 10 µL of ethyl decanoate (8.63 mg/L) were added using a CTC automatic sampler. The sample was equilibrated at 600 r/min and 60 °C for 10 min. Then, the headspace vials were exposed to a 50/30 μm divinylbenzene/Carboxen/polydimethylsiloxane (DVB/CAR/PDMS) fiber, and adsorption was carried out for 30 min. Finally, desorption was carried out in the GC inlet at 250 °C for 10 min for GC-MS identification. The Q-TOF/MS automatically performs calibration according to a preset program before the analysis of each sample to ensure instrument stability. Quality control (QC) was prepared by mixing equal amounts of all samples and were used to evaluate system stability and data repeatability. QC samples, blank samples, and mid-concentration mixed standards were run at regular intervals during the analysis (every 5 tea samples).

An Agilent GC × GC-Q-TOF-MS (Agilent, Santa Clara, CA, USA) with an olfactometric detection port (Gerstel, Mülheim an der Ruhr, Germany) was utilized in this study. The volatiles of each sample were analyzed through a separation process employing both a one-dimensional HP-5MS column and a two-dimensional DB-17MS column, both located in the same column oven. The oven temperature program as follows: initial hold at 40 °C for 1 min, increased to 180 °C at 4 °C/min, then raised to 250 °C at 20 °C/min, and held for 1 min. The split ratio and the modulation period were 10:1 and 4 s, respectively. Mass spectrometer conditions: mass scan range, 45–500 amu; electron ionization, 70 eV; quadrupole temperature, 150 °C; and ion source temperature, 200 °C.

In 2D mode, the volatiles entering from the first-dimensional column were split into two equal parts, one part was directed to the olfactory detection port (ODP), while the other part passed through the second-dimensional column and the modulator before entering the mass spectrometer detector. The olfactory analysis was conducted by three experienced sensory evaluators. During the sniffing process, the expert recorded the retention time, aroma properties and intensity of the perceived volatile compounds. Aroma active compounds (AACs) were evaluated using a 5-point intensity method (0 for non-existent, 3 for medium, and 5 for very high intensity), with the final intensity values derived from the evaluators’ averaged scores [7].

### 2.5. Qualitative and Quantitative Analysis of Volatiles

Volatiles were identified through comparison with NIST 20 library mass spectra, authentic standards, and retention indices (RIs). RIs were obtained via a series of n-alkane lines (C7–C25) calculated for each compound. Based on our previous studies, volatile compounds were retained if they exhibited forward matches exceeding 700, reverse matches exceeding 800, and RI deviations below 15 [23]. The volatiles were quantified using a dual method combining internal standardization (ethyl decanoate) and external calibration curves. For all identified volatile compounds, 10 μL of ethyl decanoate was used as an internal standard, and quantification was carried out based on the ratio of each compound peak area to that of the internal standard. If there were compounds with commercially available standards, external calibration curves (Supplementary Table S1) were established to achieve enhanced quantitative accuracy based on our previous studies [8].

### 2.6. OAV Analysis

According to evaluation standards, the concentration of volatiles was calculated (3 g tea/150 mL water) [24]. A compound with an OAV above 1 was believed to make a significant contribution to tea’s aroma.

### 2.7. Statistical Analysis

All experiments were performed in 3 biological replicates. PCA, HCA, PLS-DA, and analysis of variance (ANOVA) were conducted by SIMCA (Umea, Sweden). The heatmap was plotted using TBtools (https://github.com/CJ-Chen/TBtools, accessed on 1 December 2024).

## 3. Results and Discussion

### 3.1. Sensory Evaluation of RCBT Samples

To study the aroma changes during RCBT processing, sensory evaluations were conducted, and the results are shown in Supplementary Table S2. Fresh leaves had a fresh aroma, shifting to floral after withering. During early fermentation, RCBT exhibited a grassy hint amidst floral notes, evolving into floral, sweet, and minty-like aromas. Finally, dried tea displayed a floral and sweet aroma with minty-like characteristics.

### 3.2. Identification and Quantification of Volatiles During RCBT Processing

**Figure 1:**
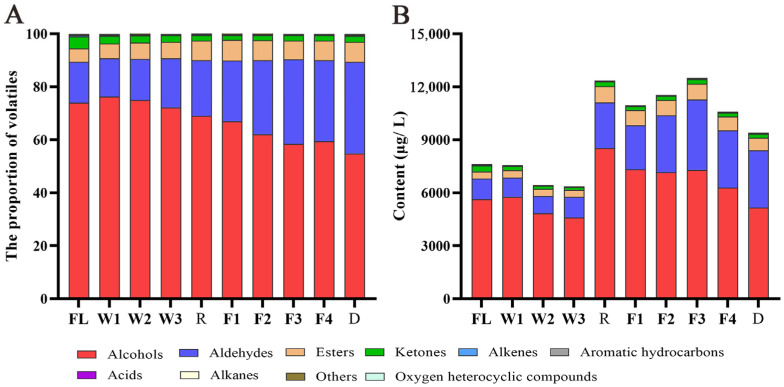
The proportion (**A**) and content (**B**) of RCBT volatiles during processing.

To investigate the aroma dynamic changes in RCBT, the volatiles were analyzed across the whole manufacturing process using SPME-GC × GC-Q-TOFMS, and the representative chromatogram of the sample is shown in Supplementary Figure S1. In total, 189 volatiles were identified during the whole processing, and they could be categorized into 10 classes, including 42 alcohols, 36 aldehydes, 31 esters, 18 ketones, 31 alkenes, 11 alkanes, 8 aromatic compounds, 2 acids, 5 oxygen heterocyclic compounds, and 5 other compounds (Supplementary Table S3). Of them, phenylethyl alcohol, linalool, (*E*)-2-hexenal, (*Z*)-3-hexen-1-ol, benzyl alcohol, (*E*)-2-hexen-1-ol, benzeneacetaldehyde, geraniol, methyl salicylate, and hexanal were the ten predominant volatiles in the RCBT processing, accounting for 84.73% of the identified compounds. Additionally, alcohols constituted the most abundant class of volatiles, accounting for 54.87–76.35% of the total identified volatiles. This was followed by aldehydes (14.41–34.58%), esters (5.18–7.78%), ketones (1.91–4.44%), alkenes (0.29–0.49%), alkanes (0.02–0.03%), aromatic hydrocarbons (0.08–0.19%), oxygen heterocyclic compounds (0.02–0.06%), acids (0–0.13%), and other compounds (0.02–0.33%) (Figure 1A). Alcohols, aldehydes, and esters were the main volatile compounds during RCBT processing, mainly contributing to the floral, sweet, and fresh aroma, which was consistent with the trend of sensory evaluation of RCBT. This result aligned broadly with the trends of our previous findings, but the proportions of alcohols and aldehydes were much higher than RCBT in earlier research [7]. The difference could be attributed to the lower response value of alcohol and aldehyde volatile compounds in GC×GC-Q-TOFMS, while previous studies mainly adopted relative quantification methods, leading to a relatively lower content of alcohol and aldehyde volatile compounds.

Alcohols are indispensable volatile compounds in black tea, mainly divided into aliphatic alcohols, aromatic alcohols, and terpene alcohols, primarily manifesting as floral, fruity, and sweet aromas [25,26]. During the processing of RCBT, the content of alcohol compounds in fresh leaves (5644.47 µg/L) decreased gradually in the withering stage (4598.08 µg/L), increased significantly after rolling (8527.31 µg/L), and fluctuated in the fermentation and drying stages. The content of alcohols in dry tea was 5158.48 µg/L. The proportion of alcohols also decreased from 73.94% in fresh leaves to 54.87% in dry tea, which was the largest reduction in the proportion of volatiles during RCBT processing (Figure 1B).

Aldehydes can be divided into terpene aldehydes, aromatic aldehydes, and aliphatic aldehydes, which mainly contribute to aroma characteristics such as fruity, green, and honey [25]. The content of aldehydes generally increased during the withering, rolling, and fermentation stages, with the most significant increase occurring during the rolling stage. In contrast, there was little to no change in the content of volatiles during the drying stage. Ultimately, the aldehyde content increased from 1171.28 µg/L in fresh leaves to 3253.12 µg/L in finished tea, with its proportion rising from 15.40% to 34.58% (Figure 1B). This is the type of compound with the largest increase in the proportion during RCBT processing. The observed transformation can be primarily attributed to the oxidative conversion of alcohol compounds, wherein the labile hydroxyl groups are readily oxidized to form corresponding aldehydes, which may subsequently undergo further oxidation to yield carboxylic acids [27].

Ester compounds constitute the third major category of volatile compounds in the RCBT processing and can be further divided into terpene esters and aromatic esters. They were closely related to the sweet, fruity, and floral aromas with high flavor-dilution factors [25]. Throughout processing, the ester content in fresh leaves (395.06 µg/L) showed minor fluctuations with little change during withering, increased rapidly after rolling (910.95 µg/L), and gradually declined during fermentation and drying (Figure 1B). Among them, the proportions of methyl salicylate and methyl geranate were pretty high.

Ketone compounds generally have a subtle aroma and contribute to the fragrance of black tea primarily through their cyclic structures, which typically manifest as floral and fruity aroma, mainly including (*Z*)-Jasmone and (*E*)-*β*-ionone. During processing, the content of ketone compounds in fresh leaves (338.18 µg/L) decreased significantly by the end of withering (158.91 µg/L), then increased rapidly after rolling (258.55 µg/L), fluctuated during fermentation, and stabilized during the drying stage. Additionally, the proportions of alkenes, aromatic compounds, acids, alkanes, and hetero-oxygen compounds during the RCBT processing were all below 0.50%. Among these, the contents of alkenes, aromatic compounds, and alkanes decreased significantly by the end of withering and then increased rapidly during the rolling stage (Figure 1B).

### 3.3. Multivariate Statistical Analysis During RCBT Processing

**Figure 2:**
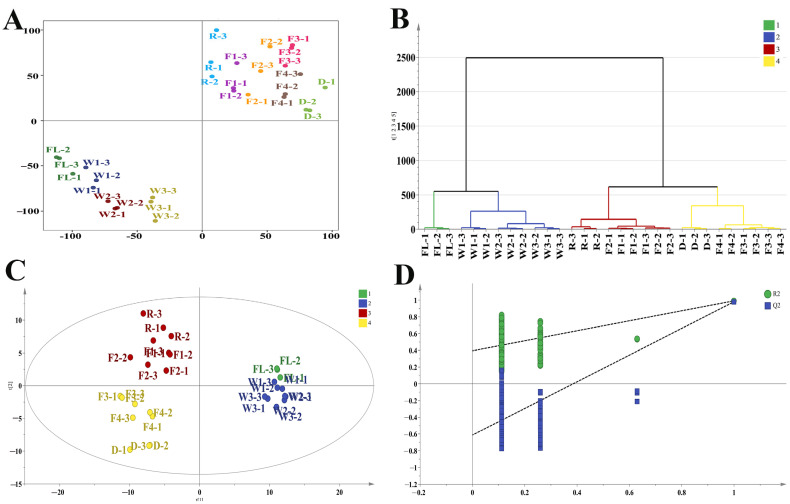
Multivariate statistical analysis of volatiles during RCBT processing. (**A**) Rotated PCA analysis, (**B**) HCA analysis, (**C**) PLS-DA analysis, and (**D**) cross-validation test.

**Figure 3:**
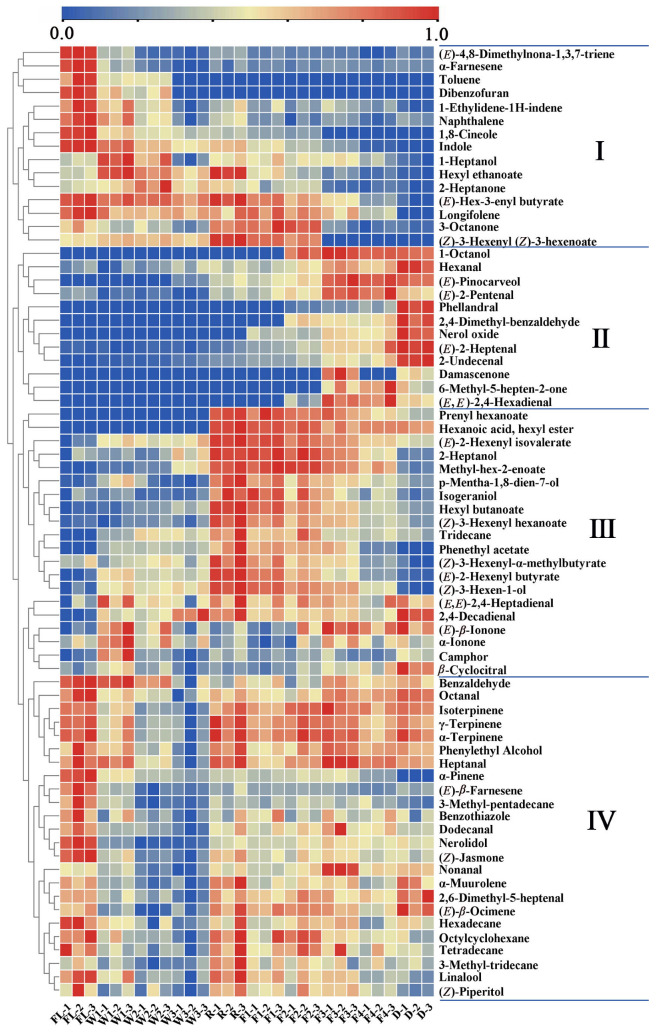
Cluster heatmap of differential metabolites (The compounds were clustered into four groups (I–IV)).

To better understand the dynamics of volatile components during RCBT processing, multivariate statistical analyses were conducted on all samples. The results of rotated PCA (PC1, 32.3%; PC2, 31.9%) (Figure 2A) and HCA (Figure 2B) demonstrated the changes in aroma of RCBT based on volatile content could be categorized into four stages: the first stage, fresh leaves (FL); the second stage, withering4 h to the end of withering(W1–W3); the third stage, rolling to 3 h of fermentation (R-F2); and the fourth stage, fermentation 4.5 h to the end of drying (F3-D). The content of volatiles in fresh leaves began to change slowly during the withering stage, showed significant changes during the rolling stage, and changed relatively little from the late fermentation to the dried tea stage. To explore the differential volatile compounds during four stages of RCBT processing, PLS-DA was performed based on the concentrations of all volatile compounds. The PLS-DA model (R2Y = 0.877, R2Y = 0.984, and Q2 = 0.954) revealed significant changes during the RCBT processing (Figure 2C). Meanwhile, the permutation test (Figure 2D) verified the model’s reliability and predictive accuracy. Seventy-one compounds were identified as key differential markers during RCBT processing (VIP > 1, *p* < 0.05). The heatmap indicated that these differential compounds could be divided into four groups based on their changing trends (Figure 3). Compounds in Group I predominantly showed a continuous decreasing trend during the processing. Most compounds in Group II exhibited slow increases during the withering and rolling stages, followed by rapid growth during the fermentation and drying stages. Compounds in Group III generally displayed an increasing trend initially, followed by a decrease. In Group IV, most compounds decreased during withering, increased during the rolling stage, and then declined again in the later fermentation and drying stages. However, the reasons for their specific changes need to be further explored.

### 3.4. Key Odorants Analysis During RCBT Processing

The aroma impact of volatiles in tea is dictated by their OAV rather than concentration. Generally, compounds with an OAV ≥ 1 are regarded as significant contributors to aroma, while those with an OAV between 0.1 and 1 are viewed as aroma modifiers [4,28]. According to quantitative results during the RCBT processing and combined with references and related websites (https://www.vcf-online.nl/VcfHome.cfm, accessed on 1 December 2024), we calculated the OAV of 85 volatiles; the results are shown in Supplementary Table S4. Among them, a total of 28 volatile compounds with an OAV ≥ 1 contributed to the aroma of RCBT (Table 1), including 14 aldehydes(heptanal, (Z)-4-heptenal, hexanal, octanal, (*E*,*E*)-2,4-heptadienal, benzeneacetaldehyde, nonanal, (2*E*,6*Z*)-nona-2,6-dienal, (2*E*)-non-2-enal, 2,4-nonadienal, neral, citral, (2*E*,4*Z*)-deca-2,4-dienal, and 2,4-decadienal); 8 alcohols (1-heptanol, 2-heptanol, (*E*)-2-hexen-1-ol, 1-octen-3-ol, linalool, (Z)-3-hexen-1-ol, phenylethyl alcohol, and geraniol); 4 ketones (*β*-damascenone, α-ionone, (*Z*)-jasmone, and (*E*)-*β*-ionone); and 2 esters (methyl salicylate and methyl geranate). These compounds contain the key aroma components of minty-like RCBT identified by recombination and omission tests, thereby validating the accuracy of previous research findings [8]. According to the synthetic pathway of endogenous volatiles in tea, the key aroma compounds in RCBT processing are mainly divided into fatty acid-derived volatiles (FADVs), volatile terpenes (VTs), amino acid-derived volatiles (AADs), and carotenoid-derived volatiles (CDVs) [9,29,30]. The total content of FADVS increased significantly at the end of withering and after rolling, and then it decreased significantly during fermentation and drying. The total amounts of VTs and AADVs gradually decreased during the withering stage but significantly increased after rolling. CDVs initially increased and decreased during the withering stage, and they fluctuated during the fermentation and drying stages.

FADVs are widely present and abundant in plants, typically originating from fatty acids such as α-linolenic acid and linoleic acid, contributing to the fresh and green scent of tea infusion [9,30]. During the RCBT processing, 17 key FADVs with OAVs exceeding 1 were identified. Interestingly, the content of most alcohol compounds significantly increased during withering and rolling, but the levels of most alcohols significantly declined during fermentation and drying, including (*Z*)-3-hexen-1-ol, 1-heptanol, 2-heptanol, and (*E*)-2-hexen-1-ol. Unlike alcohols, the content of most aldehyde compounds significantly increased at the end of the fermentation stage, such as (*E*,*E*)-2,4-hexadienal, nonanal, (2*E*)-non-2-enal, (2*E*,6*Z*)-nona-2,6-dienal, and 2,4-nonadienal, while (2*E*,4*Z*)-deca-2,4-dienal and 2,4-decadienal showed a decreasing trend. The above results were basically similar to the previous research results of black tea processing [9,10]. (2*E*,6*Z*)-Nona-2,6-dienal and (2*E*)-non-2-enal were identified as key contributors to the RCBT’s minty-like aroma in our previous study [8]. During black tea manufacturing, the rolling step promotes thorough mixing of these enzymes with lipids. Although lipoxygenase (LOX) and hydroperoxide lyase (HPL) maintain elevated enzymatic activity, alcohol dehydrogenase (ADH) undergoes rapid activity degradation during fermentation. The sustained catalytic action of LOX and HPL promotes continuous conversion of fatty acids to volatile aldehydes. However, the diminished ADH activity restricts the subsequent reduction of these aldehydes to their corresponding alcohols, resulting in substantial aldehyde accumulation and concomitant alcohol reduction [9,26].

VTs are important secondary metabolites in plants and play a significant role in food flavor, largely contributing floral and fruity notes [31]. VTs are primarily biosynthesized through two metabolic pathways in the cytoplasm: the mevalonate (MVA) pathway and the methylerythritol phosphate (MEP) pathway [31]. In our study, five terpenoids with OAV exceeding 1 were identified: geraniol, linalool, citral, neral, and methyl geranate. Among these, two of the monoterpene alcohols (linalool and geraniol) were particularly significant in establishing RCBT’s characteristic floral and sweet aroma. It was evident that these two compounds exhibited rapid increases after rolling, followed by fluctuating decreases during fermentation. Similarly, neral, citral, and methyl geranate all increased rapidly after rolling and fluctuated up during fermentation. This pattern can be attributed to the initial presence of geraniol and linalool as glycosidically bound volatiles (GBVs) in fresh leaves, where rolling-induced cellular disruption facilitates glycosidase–GBV interaction, thereby enhancing the hydrolytic release of these volatile alcohols.

Amino acids serve dual functional roles in tea, acting as both crucial taste determinants and essential precursors for aroma compound biosynthesis [32]. This study identified three amino acid derivatives with OAV > 1 (benzeneacetaldehyde, phenylethyl alcohol, and methyl salicylate). Phenylacetaldehyde and phenylethyl alcohol derived from phenylalanine exhibited rapid increases after the rolling process, followed by fluctuating rises during fermentation. The content of methyl salicylate, another phenylalanine derivative with a minty scent, increased significantly after rolling but decreased during fermentation and drying [33]. As a key aroma active compound in congou black tea, methyl salicylate has been identified as a primary contributor to the specific minty-like attributes of RCBT in previous studies [8,34].

CDVs are derived from a range of carotenoids, primarily formed during the fermentation and drying stages through both enzymatic reactions and non-enzymatic oxidation pathways, serving a crucial role in shaping distinctive aroma profiles [9,26]. During the processing of RCBT, three CDVs with OAV > 1 were identified, namely α-ionone, *β*-damascenone, and (*E*)-*β*-ionone. α-Ionone and (*E*)-*β*-ionone showed an increasing trend during the fermentation phase. These compounds have a significant impact on the floral and sweet aromas of RCBT despite their low concentrations, primarily due to their low odor thresholds, with (*E*)-*β*-ionone exhibiting the highest OAV among them. Therefore, fermentation plays an essential role in the formation of floral and sweet aromas in RCBT.

### 3.5. The Change Profile of AACs During RCBT Processing

The application of GC-O technology proves highly effective in pinpointing the specific aroma-contributing compounds found within complicated sample environments [21]. To further explore the dynamic changes in AACs, all samples of RCBT at different processing stages were detected by GC-O. Twenty-five aroma-active compounds with properties and intensity were identified by comparing with the corresponding standard (Table 2). The common attributes of these aroma compounds were floral, fruity, sweet, green, minty-like, and roasted, the same as the main aroma attributes of RCBT sensory evaluation [8]. It was worth noting that five AACs ((*E*,*E*)-2,4-hexadienal, benzyl acetate, methyl salicylate, ethyl pelargonate, and *β*-damascenone) were not detected by GC-O in fresh leaves, suggesting that processing has a pivotal effect on the aroma formation of RCBT. In addition, five aroma substances with high aroma intensity (AI ≥ 3), namely benzeneacetaldehyde, geraniol, linalool, (*E*)-*β*-ionone, and (2*E*,6*Z*)-nona-2,6-dienal, were considered to be key AACs. Among them, linalool (AI = 4.17) and (2*E*,6*Z*)-nona-2,6-dienal (AI = 4.17) possessed the highest aroma intensity, while linalool and geraniol presented the highest aroma dilution factor in RCBT’s minty-like scent [8]. Linalool and geraniol play an important role in the RCBT’s floral and sweet scent, while (2*E*,6*Z*)-nona-2,6-dienal makes a significant contribution to the formation of the minty-like aroma in RCBT [8]. Interestingly, the intensity of aroma-active compounds with floral, fruity, and sweet aroma attributes fluctuated and increased from fresh leaves to finished tea, such as (*E*)-*β*-ionone, *β*-damascenone, citral, geraniol, neral, and benzeneacetaldehyde. Similarly, this pattern was also observed in minty-like compounds, including methyl salicylate, methyl geranate, (2*E*)-non-2-enal, and (2*E*,6*Z*)-nona-2,6-dienal. The intensity changes in these AACs during RCBT processing were also generally consistent with the trend of their OAV values. Eventually, under the action of the black tea-processing procedures, the original characteristics of the fresh leaves, which were dominated by high-intensity aroma attributes, such as grass, green, fresh, and mushrooms, gradually change to the characteristics of the dried tea, which were dominated by high-intensity aroma attributes, such as floral, sweet, and minty-like aroma.

## 4. Conclusions

This study systematically investigated the aroma evolution of minty-like RCBT during processing, using sensory evaluation and GC × GC-O-Q-TOF-MS techniques. Sensory evaluation revealed a characteristic transition from a fresh aroma to a floral, sweet, and minty-like aroma during manufacturing. In addition, among 189 identified volatile compounds, alcohols constituted the predominant class (>50%), with 71 compounds identified as key differential markers throughout manufacturing stages. Aroma analysis demonstrated 28 odor-active compounds with OAV > 1 as primary contributor to RCBT’s aroma. GC-O analysis revealed 25 aroma-active compounds during RCBT processing. Notably, the characteristic minty-like odorants in RCBT are primarily generated from the metabolic pathways of the MEP and MVA, lipid oxidation, and phenylalanine. These findings provide valuable theoretical foundations for optimizing RCBT processing parameters and enhancing the quality.

## Figures and Tables

**Table 1:** OAVs of key odorants during RCBT processing.

Odorants	Thresholds	Attributes	Pathway	FL	W1	W2	W3	R	F1	F2	F3	F4	D
Hexanal	4.5	Fat, sweet	FADVs	65.88 ± 6.50 fg	58.38 ± 10.69 g	64.68 ± 3.84 fg	77.39 ± 9.58 de	81.73 ± 6.87 de	75.85 ± 4.77 ef	90.77 ± 6.58 cd	117.13 ± 5.71 b	102.96 ± 8.10 c	151.03 ± 12.77 a
(*Z*)-4-Heptenal	0.06	Baked potatoes		69.40 ± 16.96 cde	100.25 ± 32.72 abcde	71.55 ± 28.85 bcde	60.44 ± 26.98 e	127.11 ± 25.48 a	107.18 ± 12.80 abcd	111.91 ± 7.91 abc	119.38 ± 12.13 a	114.00 ± 35.05 ab	65.65 ± 15.93 de
Heptanal	10	Citrus, fat, green		7.08 ± 1.49 bc	6.91 ± 0.52 bc	4.40 ± 0.35 f	2.81 ± 0.47 g	7.66 ± 0.72 ab	4.93 ± 0.27 ef	6.01 ± 0.15 cd	8.58 ± 0.13 a	6.71 ± 0.30 bcd	5.77 ± 0.27 de
Octanal	0.7	Fat, green, nut		18.70 ± 5.63 a	7.97 ± 1.66 def	5.84 ± 0.53 ef	3.97 ± 2.91 f	9.86 ± 1.83 cde	7.49 ± 0.81 def	7.23 ± 2.18 def	13.37 ± 1.40 bc	11.24 ± 1.39 cd	15.96 ± 1.64 ab
(*E*,*E*)-2,4-Heptadienal	60	Green fruity		1.71 ± 0.25 c	2.54 ± 0.32 c	2.10 ± 0.07 c	1.89 ± 0.28 c	2.53 ± 0.36 c	2.26 ± 0.20 c	2.33 ± 0.22 c	2.30 ± 0.09 a	2.10 ± 0.45 a	2.31 ± 0.22 b
Nonanal	1	Floral, fruity		85.35 ± 4.74 c	70.99 ± 7.97 d	49.57 ± 4.01 e	44.40 ± 8.82 e	87.47 ± 10.88 c	71.32 ± 2.28 d	90.84 ± 10.44 bc	179.68 ± 10.53 a	91.41 ± 8.39 bc	103.72 ± 2.97 b
(*E*,*Z*)-2,6-Nonadienal	0.0045	Fresh, cucumber		/	/	/	57.59 ± 1.64 e	3774.45 ± 508.59 d	3785.8 ± 245.93 d	4635.61 ± 811.72 c	6084.96 ± 160.27 b	5768.37 ± 334.72 b	7239.38 ± 461.05 a
(*E*)-2-Nonenal	0.08	Fresh, cucumber-like		/	/	/	6.22 ± 1.47 e	78.30 ± 11.54 d	76.07 ± 3.20 d	166.46 ± 27.93 c	190.09 ± 10.98 ab	181.79 ± 4.38 bc	202.17 ± 7.78 a
2,4-Nonadienal	0.1	Fat, green		8.78 ± 2.27 f	12.39 ± 1.65 f	18.66 ± 0.87 de	24.08 ± 2.78 bc	17.31 ± 1.57 e	21.72 ± 1.62 cde	21.00 ± 4.66 cde	22.92 ± 1.58 bce	26.78 ± 3.32 b	38.66 ± 1.62 a
(*E*,*Z*)-2,4-Decadienal	0.04	Fat, flower, fried		1.42 ± 0.24 e	3.76 ± 0.72 c	2.45 ± 0.26 d	3.75 ± 0.43 c	5.99 ± 0.59 ab	5.23 ± 0.35 b	5.78 ± 0.73 ab	6.37 ± 0.50 a	3.96 ± 0.46 c	3.84 ± 0.63 c
2,4-Decadienal	0.3	Fat, sweet		0.37 ± 0.03 d	0.90 ± 0.11 bc	0.84 ± 0.08 c	1.36 ± 0.16 a	1.34 ± 0.17 a	1.04 ± 0.10 b	0.98 ± 0.05 bc	1.03 ± 0.05 b	0.82 ± 0.08 c	1.47 ± 0.08 a
(Z)-3-Hexen-1-ol	70	Grass, green, herb		6.29 ± 1.07 e	12.87 ± 1.33 d	11.77 ± 0.60 d	13.42 ± 1.46 d	25.85 ± 2.46 a	22.53 ± 0.98 b	18.79 ± 1.29 c	16.98 ± 0.29 c	11.56 ± 0.49 d	5.68 ± 0.29 e
(*E*)-2-Hexen-1-ol	100	Fat, fruity, geranium		0.31 ± 0.05 g	1.49 ± 0.16 f	1.16 ± 0.09 fg	2.58 ± 0.27 e	12.14 ± 1.25 a	11.48 ± 0.36 ab	10.80 ± 0.97 b	10.73 ± 0.35 b	9.68 ± 0.85 c	3.86 ± 0.25 d
2-Heptanol	70	Coconut, fried		1.23 ± 0.34 d	1.24 ± 0.10 d	1.16 ± 0.19 d	1.93 ± 0.36 c	3.53 ± 0.13 a	3.55 ± 0.26 a	3.40 ± 0.42 a	2.69 ± 0.17 b	1.81 ± 0.12 c	1.08 ± 0.03 d
1-Heptanol	5.6	Green, putrid, wood		0.37 ± 0.05 cd	1.56 ± 0.22 a	0.93 ± 0.40 b	0.11 ± 0.06 d	0.52 ± 0.11 c	0.54 ± 0.17 c	0.44 ± 0.07 c	0.51 ± 0.05 c	0.26 ± 0.09 cd	0.07 ± 0.03 d
1-Octen-3-ol	1	Earth, mushroom,		49.97 ± 2.94 d	36.18 ± 5.45 e	32.66 ± 2.17 e	34.11 ± 4.64 e	79.19 ± 7.10 a	72.43 ± 3.14 ab	64.59 ± 5.69 bc	75.76 ± 10.69 a	56.89 ± 6.08 cd	61.39 ± 6.11 c
(*Z*)-Jasmone	1.9	Floral, like jasmine		160.88 ± 12.61 a	95.25 ± 12.21 c	70.53 ± 3.15 d	65.44 ± 7.34 d	118.34 ± 10.18 b	93.71 ± 5.86 c	98.86 ± 14.58 c	106.27 ± 3.76 bc	90.77 ± 4.48 c	89.49 ± 7.53 c
Neral	100	Citrus, fruit, lemon	VTs	0.24 ± 0.03 e	0.22 ± 0.01 e	0.18 ± 0.01 e	0.28 ± 0.03 e	1.35 ± 0.12 d	1.43 ± 0.09 d	1.63 ± 0.19 c	2.11 ± 0.08 a	1.90 ± 0.05 b	1.36 ± 0.07 d
Citral	1	Fruity, like lemon		10.14 ± 1.48 e	8.91 ± 0.83 e	6.82 ± 0.74 e	12.69 ± 1.62 e	75.27 ± 6.98 d	79.51 ± 5.42 d	91.21 ± 11.00 c	119.39 ± 4.76 a	107.18 ± 3.14 b	75.74 ± 4.35 d
Linalool	6	Floral, fruity		243.34 ± 21.25 a	203.48 ± 27.22 b	165.88 ± 8.01 c	157.93 ± 18.15 c	244.32 ± 21.88 a	209.66 ± 11.10 b	197.97 ± 17.48 b	205.16 ± 3.34 b	168.53 ± 7.17 c	161.75 ± 11.95 c
Geraniol	7.5	Floral, lemon peel		46.33 ± 1.71 b	53.14 ± 3.20 b	39.83 ± 3.07 b	43.47 ± 4.65 b	96.75 ± 10.34 a	91.18 ± 6.17 a	93.56 ± 8.37 a	96.21 ± 16.70 a	91.99 ± 1.02 a	95.09 ± 9.20 a
Methyl geranate	21.44	Fruity, fresh		2.49 ± 0.21 f	1.41 ± 0.08 g	1.05 ± 0.09 g	1.67 ± 0.22 g	5.24 ± 0.49 e	5.94 ± 0.12 d	6.89 ± 0.68 c	8.09 ± 0.32 a	7.56 ± 0.31 ab	6.96 ± 0.58 bc
Benzeneacetaldehyde	4	Fruity, floral, honey	AADVs	/	/	/	/	75.32 ± 11.86 e	111.31 ± 6.13 d	164.66 ± 16.05 c	199.74 ± 4.37 b	165.54 ± 9.20 c	234.36 ± 17.79 a
Phenylethyl Alcohol	1000	Floral, fruit, honey		1.64 ± 0.25 a	1.48 ± 0.11 abc	1.26 ± 0.10 cd	1.06 ± 0.11 d	1.68 ± 0.14 a	1.38 ± 0.07 bc	1.49 ± 0.16 ab	1.70 ± 0.05 a	1.53 ± 0.06 ab	1.52 ± 0.07 ab
Methyl salicylate	40	Fresh, mint		7.07 ± 0.64 c	8.04 ± 0.60 c	7.77 ± 0.56 c	7.75 ± 0.85 c	17.27 ± 1.30 a	15.9 ± 0.75 a	15.98 ± 1.72 a	16.17 ± 0.39 a	14.33 ± 0.52 b	12.97 ± 0.67 b
Damascenone	0.002	Floral, fruit, honey	CDVs	/	/	/	/	/	/	/	697.39 ± 162.84 a	/	343.21 ± 66.50 b
α-Ionone	0.4	Floral, fruit,		7.58 ± 0.70 bcd	10.03 ± 0.77 a	8.37 ± 1.14 bc	7.77 ± 0.87 bc	7.07 ± 0.57 cd	6.18 ± 0.33 d	7.15 ± 1.24 cd	8.92 ± 0.70 ab	7.72 ± 0.80 bcd	7.75 ± 0.76 bcd
(*E*)-*β*-Ionone	0.007	Floral, sweet		2938.01 ± 174.56 e	4520.28 ± 435.7 ab	3952.33 ± 590.46 bc	3225.32 ± 354.67 cde	3272.72 ± 323.27 cde	3043.48 ± 164.9 de	3809.23 ± 691.13 bcd	4763.08 ± 219.17 a	4289.59 ± 344.18 ab	4453.36 ± 519.56 ab

Thresholds: Odor thresholds in water were obtained from the published literature or relevant websites. Different letters in the same line indicate significant differences at *p* < 0.05.

**Table 2:** GC-O analysis of RCBT during processing.

Compounds	Odor Description	FL	W1	W2	W3	R	F1	F2	F3	F4	D
Hexanal	Fresh, cut grass	2.00 ± 0.00 a	2.33 ± 0.47 a	2.67 ± 0.47 a	2.67 ± 0.47 a	2.67 ± 0.47 a	2.67 ± 0.47 a	2.67 ± 0.47 a	2.83 ± 0.62 a	2.33 ± 0.47 a	2.33 ± 0.62 a
(*Z*)-3-Hexen-1-ol	Grass, green	1.00 ± 0.00 a	1.33 ± 0.47 a	1.33 ± 0.47 a	1.67 ± 0.47 a	1.67 ± 0.47 a	1.33 ± 0.47 a	1.33 ± 0.47 a	1.33 ± 0.47 a	1.33 ± 0.47 a	1.00 ± 0.00 a
(*Z*)-4-Heptenal	Baked potatoes	1.67 ± 0.47 ab	1.83 ± 0.62 ab	1.83 ± 0.62 ab	1.33 ± 0.47 b	2.33 ± 0.47 ab	2.67 ± 0.62 a	2.17 ± 0.47 ab	2.17 ± 0.24 ab	2.67 ± 0.47 a	1.67 ± 0.47 ab
(*E*,*E*)-2,4-Hexadienal	Fat, green	/	/	/	/	/	1.33 ± 0.47 b	1.67 ± 0.47 ab	2.00 ± 0.00 a	2.33 ± 0.47 a	2.33 ± 0.47 a
1-Octen-3-ol	Mushroom-like	2.33 ± 0.47 a	1.83 ± 0.24 a	2.00 ± 0.00 a	2.00 ± 0.82 a	2.67 ± 0.47 a	2.33 ± 0.47 a	1.67 ± 0.47 a	2.67 ± 0.47 a	2.17 ± 0.24 a	2.33 ± 0.47 a
6-Methyl-5-hepten-2-one	Mushroom, pepper	1.67 ± 0.47 b	1.67 ± 0.47 b	1.67 ± 0.47 b	1.67 ± 0.47 b	2.33 ± 0.47 ab	2.33 ± 0.47 ab	1.83 ± 0.24 b	2.17 ± 0.24 ab	2.00 ± 0.00 ab	3.00 ± 0.82 a
Benzeneacetaldehyde	Floral, honey	1.33 ± 0.47 c	1.67 ± 0.47 bc	2.00 ± 0.82 abc	2.00 ± 0.82 abc	2.00 ± 0.82 abc	2.33 ± 0.47 abc	2.33 ± 0.47 abc	2.83 ± 0.62 ab	2.50 ± 0.41 abc	3.17 ± 0.62 a
Linalool oxide II	Roasted, sweet	1.67 ± 0.47 a	1.67 ± 0.47 a	1.67 ± 0.47 a	1.67 ± 0.47 a	1.33 ± 0.47 a	1.67 ± 0.47 a	1.50 ± 0.41 a	1.67 ± 0.47 a	1.67 ± 0.47 a	2.00 ± 0.41 a
Methyl benzoate	Fresh, fruity	1.67 ± 0.47 a	1.67 ± 0.47 a	2.00 ± 0.82 a	2.33 ± 0.47 a	2.00 ± 0.82 a	2.33 ± 0.47 a	2.33 ± 0.47 a	1.67 ± 0.47 a	2.00 ± 0.00 a	2.00 ± 0.00 a
Linalool	Floral, sweet	3.83 ± 0.24 a	3.67 ± 0.47 a	3.67 ± 0.47 a	3.33 ± 0.47 a	3.67 ± 0.47 a	3.67 ± 0.47 a	3.67 ± 0.47 a	3.67 ± 0.47 a	4.17 ± 0.24 a	4.17 ± 0.24 a
Phenylethyl Alcohol	Floral, fruit, honey	1.33 ± 0.47 a	1.33 ± 0.47 a	1.33 ± 0.47 a	1.33 ± 0.47 a	1.67 ± 0.47 a	1.33 ± 0.47 a	1.33 ± 0.47 a	1.17 ± 0.24 a	1.17 ± 0.24 a	1.33 ± 0.47 a
(2*E*,6*Z*)-Nona-2,6-dienal	Fresh, cucumber	1.67 ± 0.47 b	2.33 ± 0.47 b	2.33 ± 0.47 b	2.33 ± 0.47 b	3.33 ± 0.47 a	3.33 ± 0.47 a	3.33 ± 0.47 a	4.00 ± 0.00 a	3.50 ± 0.41 a	4.17 ± 0.24 a
(2*E*)-Non-2-enal	Fresh, cucumber-like	1.00 ± 0.00 d	1.00 ± 0.00 d	1.33 ± 0.47 cd	1.67 ± 0.47 cd	2.00 ± 0.00 bc	2.00 ± 0.00 bc	2.67 ± 0.47 ab	2.67 ± 0.47 ab	2.50 ± 0.41 ab	2.83 ± 0.24 a
Benzyl acetate	Fruity, fresh	/	/	/	/	1.00 ± 0.00 a	1.33 ± 0.47 a	1.33 ± 0.47 a	1.33 ± 0.47 a	1.67 ± 0.47 a	1.33 ± 0.47 a
Terpinen-4-ol	Fresh, woody	2.33 ± 0.47 a	2.33 ± 0.47 a	3.00 ± 0.00 a	3.00 ± 0.00 a	3.00 ± 0.00 a	3.00 ± 0.00 a	2.67 ± 0.47 a	3.00 ± 0.00 a	3.00 ± 0.00 a	2.50 ± 0.41 a
Methyl salicylate	Fresh, mint	/	1.33 ± 0.47 b	1.83 ± 0.24 ab	1.83 ± 0.24 ab	2.17 ± 0.24 a	2.33 ± 0.47 a	2.00 ± 0.00 ab	1.67 ± 0.47	2.17 ± 0.24 a	1.67 ± 0.47 ab
2,4-Nonadienal	Deep-fried, fat	1.00 ± 0.00 a	1.83 ± 0.24 a	1.50 ± 0.41 a	1.83 ± 0.24 a	1.33 ± 0.47 a	1.67 ± 0.47 a	1.33 ± 0.47 a	1.33 ± 0.47 a	1.33 ± 0.47 a	1.67 ± 0.47 a
Neral	Fruity, lemon	1.33 ± 0.47 b	1.33 ± 0.47 b	1.33 ± 0.47 b	2.00 ± 0.00 ab	2.67 ± 0.47 a	2.00 ± 0.00 ab	2.33 ± 0.47 a	2.67 ± 0.47 a	2.33 ± 0.47 a	2.50 ± 0.41 a
Geraniol	Floral, like rose	2.50 ± 0.41 b	2.83 ± 0.62 ab	3.17 ± 0.24 ab	3.17 ± 0.24 ab	3.50 ± 0.41 ab	3.50 ± 0.41 ab	3.67 ± 0.47 a	3.50 ± 0.41 ab	3.33 ± 0.47 ab	3.67 ± 0.47 a
Citral	Fruity, like lemon	1.33 ± 0.47 c	1.50 ± 0.41 bc	1.50 ± 0.41 bc	2.00 ± 0.00 abc	2.17 ± 0.24 ab	2.17 ± 0.24 ab	2.17 ± 0.24 ab	2.17 ± 0.24 ab	2.50 ± 0.41 a	2.17 ± 0.24 ab
Ethyl nonanoate	Fresh, fruity	/	/	0.67 ± 0.47 a	0.67 ± 0.47 a	0.67 ± 0.47 a	0.33 ± 0.47 a	0.33 ± 0.47 a	0.33 ± 0.47 a	0.67 ± 0.47 a	0.33 ± 0.47 a
Methyl geranate	Fruit, fresh	1.17 ± 0.24 b	1.33 ± 0.47 ab	1.33 ± 0.47 ab	1.33 ± 0.47 ab	1.17 ± 0.24 b	1.17 ± 0.24 b	1.17 ± 0.24 b	2.00 ± 0.00 a	1.50 ± 0.41 ab	1.83 ± 0.24 ab
*β*-Damascenone	Sweet, honey	/	/	/	/	/	/	/	2.33 ± 0.47 a	1.67 ± 0.47 b	2.33 ± 0.47 a
(*Z*)-Jasmone	Floral, sweet	1.83 ± 0.24 a	1.33 ± 0.47 ab	1.33 ± 0.47 ab	1.33 ± 0.47 ab	1.00 ± 0.00 b	1.17 ± 0.24 ab	1.17 ± 0.24 ab	1.00 ± 0.00 b	1.33 ± 0.47 ab	1.00 ± 0.00 b
(*E*)-*β*-Ionone	Floral, sweet	2.33 ± 0.47 a	2.67 ± 0.47 a	2.33 ± 0.47 a	2.50 ± 0.41 a	2.33 ± 0.47 a	2.33 ± 0.47 a	2.67 ± 0.47 a	3.33 ± 0.47 a	3.00 ± 0.00 a	3.00 ± 0.00 a

Different letters in the same line indicate significant differences at *p* < 0.05.

## Data Availability

The original contributions presented in the study are included in the article/Supplementary Materials. Further inquiries can be directed to the corresponding authors.

## Supplementary Materials

The following supporting information can be downloaded at: https://www.mdpi.com/article/10.3390/foods14111941/s1, Figure S1: Representative total ion chromatogram of RCBT process sample; Table S1: Quantitative standard curve of RCBT volatile compounds; Table S2: Result of sensory evaluation in minty-like RCBT; Table S3: The content of volatiles during RCBT processing; Table S4: OAV of all volatiles in RCBT processing.
