# Supplementary material for: Characterization of Key Odorants During Processing of Minty-like Aroma ‘Rucheng Baimaocha’ Black Tea
Source: Foods. 2025 May 29;14(11):1941. doi: 10.3390/foods14111941 (PMC12154047; doi:10.3390/foods14111941)
Supplement: Supplementary file 1 [file foods-14-01941-s001.zip › foods-3657361-supplementary.pdf]

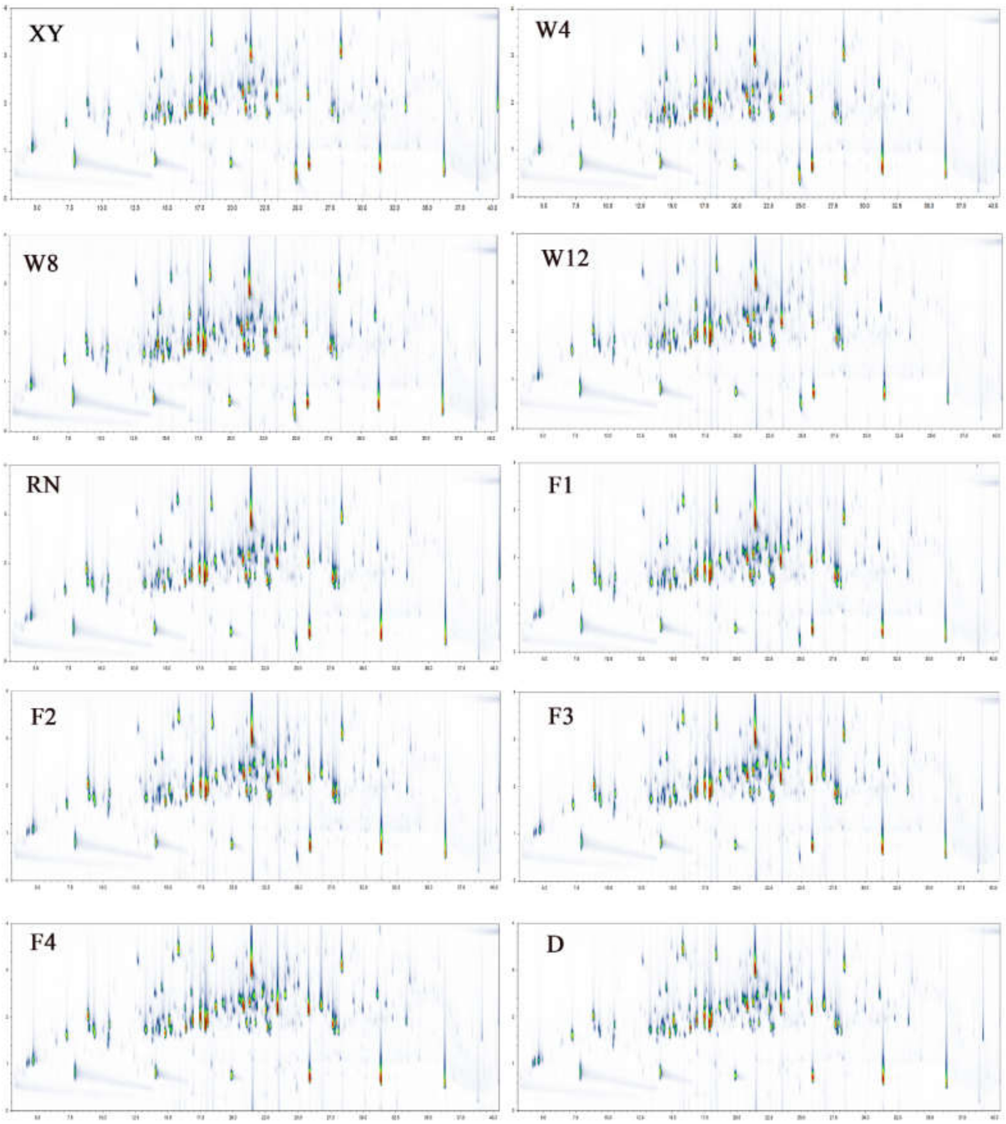


**Figure S1. Representative total ion chromatogram of RCBT process sample**
FL: fresh leaves; W4: withering 4h sample; W8: withering 8h sample; W12: withering 12h sample; R: rolling sample; F1: fermentation for 1.5h sample; F2: fermentation for 3h sample; F3: fermentation for 4.5h sample; F4: fermentation for 6h sample; D: drying sample. The following were the same.

**Table S1. Quantitative standard curve of RCBT volatile compounds**

| Compounds | Calibration curve equation | $R^2$ | Linear concentration ranges(μg/L) |
|---|---|---|---|
| Hexanal | y=0.571x+0.181 | 0.9925 | 2.00-2000.00 |
| (*Z*)-4-Heptenal | y=2.0888x+0.037 | 0.9998 | 0.20-400.00 |
| 1-Octen-3-ol | y=4.8769x+0.219 | 0.9919 | 0.10-200.00 |
| 6-Methyl-5-hepten-2-one | y=4.9371x+0.2288 | 0.9951 | 0.10-400.00 |
| Benzeneacetaldehyde | y=0.663x+0.405 | 0.9981 | 4.00-200.00 |
| Methylbenzoate | y=9.154x+0.0474 | 0.9999 | 0.10-200.00 |
| Linalool | y=2.7253x+0.6535 | 0.9936 | 0.10-2000.00 |
| Nonanal | y=5.8998x+0.7349 | 0.9985 | 0.20-400.00 |
| Phenylethyl Alcohol | y=0.2898x-0.0172 | 0.9998 | 20.00-4000.00 |
| (2*E*, 6*Z*)-Nona-2,6-dienal | y=1.9686x+0.1073 | 0.9988 | 1.00-1000.00 |
| (2*E*)-Non-2-enal | y=5.931x+0.1095 | 0.9989 | 0.04-400.00 |
| Benzyl acetate | y=8.8527x+0.0811 | 0.997 | 0.40-200.00 |
| Terpinen-4-ol | y=6.9871x+0.2527 | 0.9922 | 0.10-200.00 |
| Hexyl butanoate | y=17.337x+0.2195 | 0.9993 | 0.10-200.00 |
| Methyl salicylate | y=5.2611x+0.4255 | 0.9974 | 0.40-1000.00 |
| 2,4-Nonadienal | y=7.0081x+0.0046 | 0.9996 | 0.40-200.00 |
| Neral | y=1.6527x-0.0119 | 0.9986 | 0.40-200.00 |
| Geraniol | y=3.2765x+0.1319 | 0.9906 | 1.00-1000.00 |
| Citral | y=2.8162x+0.1652 | 0.9985 | 2.00-1000.00 |
| Methyl geranate | y=10.179x+0.5321 | 0.9994 | 0.40-400.00 |
| *β*-Damascenone | y=11.057x+0.1056 | 0.9916 | 0.40-200.00 |
| (*Z*)-Jasmone | y=3.7752x+0.1901 | 0.9985 | 1.00-2000.00 |
| *α*-Ionone | y=14.501x+0.0143 | 0.9996 | 0.40-200.00 |
| (*E*)-*β*-Ionone | y=6.4972x+0.0461 | 0.9999 | 1.00-1000.00 |
| (*E*)-2-Hexenal | y=0.4412x+0.0228 | 0.9994 | 1.00-1000.00 |
| Heptanal | y=2.1748x+0.1468 | 0.9941 | 0.10-400.00 |
| Benzaldehyde | y=2.1871x+0.3293 | 0.9989 | 1.00-1000.00 |
| (*E*)-2-Hexen-1-ol | y=0.2969x+0.0068 | 0.9999 | 10.00-2000.00 |
| 1-Heptanol | y=1.5546x+0.0214 | 0.9999 | 0.40-1000.00 |
| Benzyl alcohol | y=0.1419x-0.0292 | 0.9977 | 40.00-4000.00 |
| 2-Heptanol | y=0.6203x+0.1647 | 0.9965 | 0.20-2000.00 |
| (*Z*)-3-Hexen-1-ol | y=0.156x+0.0046 | 0.9994 | 20.00-4000.00 |
| Acetophenone | y=5.9984x+0.0239 | 0.9945 | 1.00-40.00 |
| 3-Octanone | y=71.023x-0.0157 | 0.9986 | 0.04-2.00 |
| *β*-Myrcene | y=38.335X+0.0971 | 0.9999 | 0.004-40.00 |
| 2-Butyl-furan | y=9.7039x+0.0062 | 0.9966 | 0.004-200.00 |
| Nerol | y=4.114x+0.2139 | 0.9953 | 4.00-1000.00 |

**Table S2. Result of sensory evaluation in minty-like RCBT**

| Tea Samples | Aroma attributes |
|---|---|
| FL | Fresh |
| W1 | Fresh |
| W2 | fresh |
| W3 | Fresh with floral |
| R | Floral |
| F1 | Floral with green |
| F2 | Floral with slightly minty-like |
| F3 | Floral, sweet with slightly minty-like |
| F4 | Floral, sweet with minty-like |
| D | Floral, sweet with minty-like |

**Table S3. The content of volatiles during RCBT processing**

| Compounds | Formula | CAS | RT1 (min) | RI/Nist RI | F/R | FL | W1 | W2 | W3 | R | F1 | F2 | F3 | F4 | D |
|---|---|---|---|---|---|---|---|---|---|---|---|---|---|---|---|
| **Aldehydes** | | | | | | | | | | | | | | | |
| (*E*)-2-Pentenal | C5H8O | 1576-87-0 | 6.1268 | 749/753 | 805/865 | 0.05±0.00 | 0.03±0.01 | 0.04±0.00 | 0.03±0.00 | 0.07±0.01 | 0.07±0.01 | 0.09±0.01 | 0.13±0.00 | 0.12±0.01 | 0.09±0.01 |
| Hexanal | C6H12O | 66-25-1 | 7.2577 | 800/801 | 931/948 | 296.48±29.27 | 262.70±48.09 | 291.04±17.27 | 348.26±43.11 | 367.79±30.94 | 341.32±21.47 | 408.46±29.61 | 527.07±25.68 | 463.31±36.43 | 679.63±57.46 |
| (*E*)-2-Hexenal | C6H10O | 6728-26-3 | 8.8596 | 851/851 | 833/861 | 528.65±25.20 | 457.90±65.45 | 428.53±24.70 | 581.38±66.23 | 1342.35±146.3 | 1181.63±71.76 | 1541.22±135.69 | 1829.05±25.7 | 1445.56±85.2 | 1027.02±108.01 |
| (Z)-4-Heptenal | C7H12O | 6728-31-0 | 10.4575 | 900/900 | 822/896 | 4.16±1.02 | 6.02±1.96 | 4.29±1.73 | 3.63±1.62 | 7.63±1.53 | 6.43±0.77 | 6.71±0.47 | 7.16±0.73 | 6.84±2.10 | 3.94±0.96 |
| Heptanal | C7H14O | 111-71-7 | 10.5244 | 902/901 | 883/885 | 70.81±14.90 | 69.10±5.22 | 43.95±3.49 | 28.14±4.68 | 76.63±7.21 | 49.30±2.70 | 60.13±1.50 | 85.80±1.26 | 67.1±3.04 | 57.69±2.72 |
| (*E*, *E*)-2,4-Hexadienal | C6H8O | 142-83-6 | 10.8617 | 912/911 | 834/843 | RD | RD | RD | RD | RD | RD | 0.90±0.46 | 5.25±1.05 | 4.98±2.07 | 2.26±0.23 |
| (*E*)-2-Heptenal | C7H12O | 18829-55-5 | 12.5244 | 957/958 | 896/907 | 0.06±0.00 | 0.06±0.01 | 0.07±0.00 | 0.07±0.01 | 0.08±0.01 | 0.11±0.00 | 0.13±0.01 | 0.18±0.00 | 0.20±0.04 | 0.27±0.01 |
| Benzaldehyde | C7H6O | 100-52-7 | 12.7244 | 963/962 | 906/906 | 26.82±4.41 | 25.71±4.16 | 15.12±2.49 | 4.64±3.61 | 6.08±3.99 | 8.02±2.81 | 4.00±1.79 | 13.69±2.67 | 8.73±1.35 | 11.09±2.85 |
| Octanal | C8H16O | 124-13-0 | 14.2576 | 1004/1003 | 880/922 | 13.09±3.94 | 5.58±1.17 | 4.09±0.37 | 2.78±2.03 | 6.90±1.28 | 5.25±0.57 | 5.06±1.53 | 9.36±0.98 | 7.87±0.98 | 11.17±1.15 |
| (*E*, *E*)-2,4-Heptadienal | C7H10O | 4313-03-5 | 14.5245 | 1011/1012 | 852/852 | 102.77±14.95 | 152.61±19.03 | 126.02±4.12 | 113.16±17.03 | 151.72±21.5 | 135.82±12.24 | 139.94±13.22 | 137.82±5.1 | 125.72±27.09 | 138.53±13.44 |
| 5-Ethylcyclopent-1-enecarboxaldehyde | C8H12O | 36431-60-4 | 15.391 | 1035/1040 | 794/803 | 0.13±0.01 | 0.14±0.01 | 0.14±0.02 | 0.10±0.01 | 0.07±0.01 | 0.06±0.00 | 0.05±0.00 | 0.05±0.01 | 0.06±0.01 | 0.14±0.01 |
| Benzeneacetaldehyde | C8H8O | 122-78-1 | 15.791 | 1046/1045 | 906/911 | RQ | RQ | RQ | RQ | 301.28±47.45 | 445.23±24.51 | 658.65±64.19 | 798.98±17.49 | 662.17±36.79 | 937.45±71.18 |
| 2,6-Dimethyl-5-heptenal | C9H16O | 106-72-9 | 16.1911 | 1056/1054 | 823/918 | 0.43±0.01 | 0.31±0.04 | 0.28±0.03 | 0.20±0.02 | 0.43±0.05 | 0.38±0.02 | 0.45±0.04 | 0.39±0.05 | 0.38±0.03 | 0.51±0.06 |
| (*E*)-2-Octenal | C8H14O | 2548-87-0 | 16.3242 | 1059/1060 | 903/925 | 0.53±0.03 | 0.76±0.12 | 0.83±0.15 | 0.66±0.10 | 0.98±0.18 | 1.48±0.08 | 1.48±0.28 | 1.81±0.10 | 1.79±0.25 | 2.17±0.05 |
| Nonanal | C9H18O | 124-19-6 | 18.0577 | 1106/1104 | 896/897 | 85.35±4.74 | 70.99±7.97 | 49.57±4.01 | 44.40±8.82 | 87.47±10.88 | 71.32±2.28 | 90.84±10.44 | 179.68±10.53 | 91.41±8.39 | 103.72±2.97 |
| (2*E*, 6*Z*)-Nona-2,6-dienal | C9H14O | 557-48-2 | 19.8603 | 1155/1155 | 839/882 | RD | RD | RD | 0.26±0.01 | 16.99±2.29 | 17.04±1.11 | 20.86±3.65 | 27.38±0.72 | 25.96±1.51 | 32.58±2.07 |

| | | | | | | | | | | | | | | | |
|---|---|---|---|---|---|---|---|---|---|---|---|---|---|---|---|
| (2*E*)-Non-2-enal | C9H16O | 18829-56-6 | 20.1243 | 1162/1162 | 870/901 | RQ | RQ | RQ | 0.50±0.12 | 6.26±0.92 | 6.09±0.26 | 13.32±2.23 | 15.21±0.88 | 14.54±0.35 | 16.17±0.62 |
| 2,4-Dimethyl-benzaldehyde | C9H10O | 15764-16-6 | 20.7268 | 1179/1182 | 792/835 | RD | RD | RD | RD | RD | RD | 0.14±0.01 | 0.12±0.01 | 0.13±0.02 | 0.23±0.02 |
| Decanal | C10H20O | 112-31-2 | 21.7243 | 1206/1206 | 886/886 | 3.24±0.39 | 3.17±0.30 | 3.14±0.22 | 2.22±0.52 | 3.75±0.14 | 2.88±0.31 | 3.19±0.64 | 4.39±0.86 | 3.73±0.62 | 4.12±0.29 |
| 2,4-Nonadienal | C9H14O | 5910-87-2 | 22.0578 | 1216/1214 | 856/861 | 0.88±0.23 | 1.24±0.17 | 1.87±0.09 | 2.41±0.28 | 1.73±0.16 | 2.17±0.16 | 2.1±0.47 | 2.29±0.16 | 2.68±0.33 | 3.87±0.16 |
| *β*-Cyclocitral | C10H16O | 432-25-7 | 22.3909 | 1226/1220 | 875/882 | 1.46±0.13 | 1.97±0.17 | 1.60±0.18 | 1.30±0.14 | 1.46±0.08 | 1.37±0.09 | 1.61±0.18 | 1.82±0.08 | 1.95±0.33 | 2.68±0.31 |
| Neral | C10H16O | 106-26-3 | 22.9909 | 1243/1240 | 900/901 | 23.81±2.54 | 21.7±1.43 | 18.12±1.27 | 28.19±2.78 | 135.49±11.96 | 142.75±9.29 | 162.81±18.86 | 211.13±8.15 | 190.2±5.38 | 136.3±7.46 |
| 4-(1-Methylethyl)-benzaldehyde | C10H12O | 122-03-2 | 23.0578 | 1245/1239 | 743/886 | 0.05±0.00 | 0.07±0.01 | 0.05±0.01 | 0.06±0.01 | 0.14±0.01 | 0.22±0.06 | 0.2±0.01 | 0.23±0.01 | 0.25±0.04 | 0.13±0.01 |
| *β*-Homocyclocitral | C11H18O | 472-66-2 | 23.6578 | 1262/1254 | 891/900 | 0.52±0.04 | 0.69±0.11 | 0.49±0.08 | 0.48±0.07 | 0.82±0.04 | 0.73±0.12 | 0.84±0.13 | 1.03±0.06 | 0.87±0.01 | 1.12±0.22 |
| (*E*)-2-Decenal | C10H18O | 3913-81-3 | 23.7243 | 1264/1263 | 805/869 | 0.11±0.01 | 0.34±0.06 | 0.19±0.02 | 0.22±0.06 | 0.39±0.03 | 0.41±0.06 | 0.50±0.01 | 0.62±0.06 | 0.60±0.06 | 0.78±0.11 |
| Citral | C10H16O | 5392-40-5 | 24.0575 | 1274/1273 | 894/894 | 10.14±1.48 | 8.91±0.83 | 6.82±0.74 | 12.69±1.62 | 75.27±6.98 | 79.51±5.42 | 91.21±11 | 119.39±4.76 | 107.18±3.14 | 75.74±4.35 |
| *α*-Ethylidene-benzeneacetaldehyde | C10H10O | 4411-89-6 | 24.1287 | 1276/1276 | 801/876 | RD | RD | RD | RD | RD | RD | 0.10±0.02 | 0.08±0.01 | 0.08±0.02 | 0.22±0.01 |
| Perillaldehyde | C10H14O | 2111-75-3 | 24.2575 | 1280/1274 | 900/900 | 0.35±0.03 | 0.27±0.01 | 0.20±0.02 | 0.29±0.03 | 1.77±0.15 | 1.73±0.12 | 1.71±0.24 | 1.79±0.12 | 1.56±0.07 | 1.37±0.10 |
| Phellandral | C10H16O | 21391-98-0 | 24.3284 | 1280/1276 | 835/895 | RD | RD | RD | RD | RD | RD | 0.05±0.01 | 0.08±0.01 | 0.11±0.03 | 0.41±0.02 |
| *α*-Terpinen-7-al | C10H14O | 1197-15-5 | 24.5932 | 1289/1283 | 798/813 | RD | RD | RD | RD | 0.13±0.01 | 0.09±0.01 | 0.09±0.02 | 0.10±0.00 | 0.09±0.01 | 0.11±0.01 |
| (2*E*,4Z)-Deca-2,4-dienal | C10H16O | 25152-83-4 | 24.7927 | 1295/1295 | 798/833 | 0.06±0.01 | 0.15±0.03 | 0.10±0.01 | 0.15±0.02 | 0.24±0.02 | 0.21±0.01 | 0.23±0.03 | 0.25±0.02 | 0.16±0.02 | 0.15±0.03 |
| Undecanal | C11H22O | 112-44-7 | 25.2575 | 1309/1307 | 878/906 | 0.37±0.03 | 0.32±0.05 | 0.29±0.02 | 0.19±0.04 | 0.44±0.02 | 0.37±0.06 | 0.39±0.05 | 0.51±0.16 | 0.44±0.05 | 0.35±0.02 |
| 2,4-Decadienal | C10H16O | 2363-88-4 | 25.5909 | 1319/1317 | 702/858 | 0.11±0.01 | 0.27±0.03 | 0.25±0.02 | 0.41±0.05 | 0.40±0.05 | 0.31±0.03 | 0.30±0.01 | 0.31±0.02 | 0.25±0.02 | 0.44±0.02 |
| 2-Undecenal | C11H20O | 2463-77-6 | 27.1244 | 1366/1366 | 875/811 | 0.04±0.00 | 0.06±0.00 | 0.06±0.00 | 0.08±0.01 | 0.11±0.00 | 0.13±0.00 | 0.14±0.02 | 0.21±0.02 | 0.21±0.05 | 0.38±0.01 |
| 2-Butyl-2-octenal | C12H22O | 13019-16-4 | 27.4575 | 1376/1378 | 808/822 | 0.47±0.08 | 0.31±0.02 | 0.30±0.02 | 0.34±0.05 | 0.18±0.01 | 0.14±0.02 | 0.07±0.00 | 0.07±0.02 | 0.06±0.01 | 0.09±0.01 |
| Dodecanal | C12H24O | 112-54-9 | 28.591 | 1411/1409 | 865/883 | 0.32±0.03 | 0.19±0.01 | 0.18±0.02 | 0.13±0.02 | 0.26±0.04 | 0.23±0.03 | 0.25±0.02 | 0.31±0.08 | 0.26±0.02 | 0.21±0.00 |
| **Alcohols** | | | | | | | | | | | | | | | |

| | | | | | | | | | | | | | | | |
|---|---|---|---|---|---|---|---|---|---|---|---|---|---|---|---|
| (*Z*)-2-Penten-1-ol | C5H10O | 1576-95-0 | 6.4577 | 764/768 | 861/892 | 0.10±0.01 | 0.08±0.01 | 0.09±0.01 | 0.16±0.02 | 0.35±0.06 | 0.47±0.05 | 0.52±0.02 | 0.67±0.01 | 0.56±0.03 | 0.49±0.05 |
| (*Z*)-3-Hexen-1-ol | C6H12O | 928-96-1 | 8.9909 | 855/857 | 893/895 | 440.07±75.12 | 900.72±93.39 | 823.91±41.69 | 939.22±102.30 | 1809.16±172.4 | 1576.87±68.48 | 1315.30±90.17 | 1188.25±20.02 | 809.41±34.41 | 397.43±20.61 |
| (*E*)-2-Hexen-1-ol | C6H12O | 928-95-0 | 9.3261 | 865/862 | 898/899 | 31.41±5.00 | 148.94±16.50 | 116.07±8.57 | 257.68±27.28 | 1213.84±124.67 | 1148.17±35.97 | 1080.36±97.08 | 1072.91±35.19 | 967.58±85.01 | 386.23±25.06 |
| 1-Hexanol | C6H14O | 111-27-3 | 9.3927 | 867/868 | 884/884 | 0.33±0.05 | 0.89±0.20 | 0.78±0.14 | 1.33±0.14 | 3.24±0.38 | 2.88±0.15 | 2.97±0.23 | 3.70±0.09 | 3.20±0.10 | 1.76±0.11 |
| 2-Heptanol | C7H16O | 543-49-7 | 10.4575 | 900/900 | 720/850 | 86.03±23.48 | 87.06±7.26 | 81.29±13.61 | 135.27±25.40 | 246.8±9.37 | 248.55±18.21 | 237.94±29.25 | 188±12.06 | 126.95±8.61 | 75.35±1.98 |
| 1-Heptanol | C7H16O | 111-70-6 | 12.991 | 969/970 | 843/910 | 2.06±0.31 | 8.71±1.26 | 5.23±2.24 | 0.64±0.35 | 2.92±0.61 | 3.03±0.97 | 2.47±0.41 | 2.83±0.29 | 1.43±0.48 | 0.41±0.15 |
| 1-Octen-3-ol | C8H16O | 3391-86-4 | 13.3244 | 979/980 | 715/812 | 49.97±2.94 | 36.18±5.45 | 32.66±2.17 | 34.11±4.64 | 79.19±7.10 | 72.43±3.14 | 64.59±5.69 | 75.76±10.69 | 56.89±6.08 | 61.39±6.11 |
| Benzyl alcohol | C7H8O | 100-51-6 | 15.391 | 1035/1036 | 903/906 | 1351.37±144.91 | 1292.13±56.52 | 1061.06±33.9 | 727.82±71.87 | 927.65±81.4 | 600.15±32.05 | 689.18±78.7 | 707.89±191.9 | 708.33±21.6 | 646.46±11.77 |
| *α*-Methyl-Benzenemethanol | C8H10O | 98-85-1 | 16.4587 | 1063/1061 | 892/908 | RD | 0.08±0.00 | 0.06±0.01 | 0.03±0.00 | 0.10±0.01 | 0.10±0.01 | 0.16±0.01 | 0.19±0.02 | 0.16±0.01 | 0.16±0.01 |
| (*Z*)-Thujane-4-ol | C10H18O | 15537-55-0 | 16.726 | 1070/1070 | 902/902 | 7.56±0.31 | 6.52±0.96 | 5.59±0.27 | 4.47±0.52 | 4.68±0.40 | 2.92±0.42 | 2.00±0.15 | 1.87±0.05 | 1.33±0.07 | 1.02±0.02 |
| 1-Octanol | C8H18O | 111-87-5 | 16.7951 | 1072/1070 | 762/837 | RD | RD | RD | RD | RD | RD | 0.65±0.07 | 0.79±0.05 | 0.68±0.02 | 0.65±0.03 |
| Linalool oxide Ⅰ | C10H18O2 | 5989-33-3 | 16.9242 | 1075/1074 | 889/891 | 9.16±0.57 | 9.36±1.11 | 8.48±0.53 | 11.09±1.16 | 17.68±1.67 | 15.47±0.67 | 15.41±1.78 | 16.54±0.53 | 14.55±0.59 | 14.56±0.62 |
| Linalool oxide II | C10H18O2 | 34995-77-2 | 17.5242 | 1092/1086 | 888/889 | 19.48±1.79 | 17.54±1.96 | 14.82±1.10 | 16.7±1.82 | 26.96±2.56 | 23.83±1.23 | 26.09±3.14 | 24.65±0.42 | 21.76±1.28 | 22.31±1.35 |
| Linalool | C10H18O | 78-70-6 | 17.9242 | 1103/1099 | 885/885 | 1460.02±127.5 | 1220.86±163.34 | 995.26±48.09 | 947.56±108.88 | 1465.94±131.28 | 1257.95±66.6 | 1187.84±104.88 | 1230.93±20.01 | 1011.2±43.02 | 970.48±71.72 |
| Hotrienol | C10H16O | 20053-88-7 | 18.12 | 1106/1106 | 843/919 | 2.94±0.31 | 3.95±0.89 | 2.66±0.08 | 3.26±0.34 | 5.21±0.72 | 4.67±0.31 | 4.76±0.94 | 4.73±0.35 | 4.65±0.52 | 10.61±1.17 |
| 2, 6-Dimethyl-cyclohexanol | C8H16O | 5337-72-4 | 18.2595 | 1112/1112 | 845/869 | 0.61±0.03 | 0.71±0.04 | 0.63±0.09 | 0.43±0.05 | 0.40±0.03 | 0.37±0.02 | 0.37±0.05 | 0.45±0.04 | 0.40±0.05 | 0.4±0.03 |
| Phenylethyl Alcohol | C8H10O | 60-12-8 | 18.3911 | 1116/1116 | 929/929 | 1639.47±253.02 | 1481.41±105.41 | 1256.04±100.89 | 1063.25±108.84 | 1679.43±141.44 | 1379.82±71.58 | 1494.42±160.4 | 1701.79±53.55 | 1529.93±57.06 | 1520.94±72.36 |

| | | | | | | | | | | | | | | | |
|---|---|---|---|---|---|---|---|---|---|---|---|---|---|---|---|
| (*Z*)-p-Mentha-2,8-dien-1-ol | $C_{10}H_{16}O$ | 7212-40-0 | 18.7242 | 1124/1123 | 888/894 | 5.98±0.26 | 5.44±0.68 | 4.69±0.69 | 4.39±0.46 | 8.79±0.84 | 7.68±0.81 | 8.97±1.22 | 9.30±0.45 | 9.32±0.27 | 7.85±0.70 |
| p-Menth-2-en-1-ol | $C_{10}H_{18}O$ | 29803-81-4 | 19.3908 | 1142/1140 | 868/874 | 1.05±0.08 | 0.9±0.11 | 0.81±0.05 | 0.71±0.13 | 1.69±0.09 | 1.53±0.1 | 1.42±0.22 | 1.44±0.06 | 1.21±0.05 | 1.25±0.15 |
| (*E*)-Pinocarveol | $C_{10}H_{16}O$ | 547-61-5 | 19.4603 | 1144/1139 | 897/920 | RD | RD | RD | 0.15±0.02 | 0.41±0.07 | 0.39±0.06 | 0.53±0.05 | 1.06±0.12 | 1.04±0.08 | 0.94±0.06 |
| (*Z*)-3-Nonen-1-ol | $C_9H_{18}O$ | 10340-23-5 | 19.8603 | 1155/1156 | 878/897 | RD | RD | 0.10±0.01 | 0.21±0.03 | 0.75±0.06 | 0.65±0.03 | 0.62±0.08 | 0.66±0.02 | 0.52±0.03 | 0.37±0.03 |
| Nerol oxide | $C_{10}H_{16}O$ | 1786-08-9 | 19.9265 | 1157/1154 | 808/836 | RD | RD | RD | RD | RD | 0.07±0.01 | 0.07±0.01 | 0.11±0.01 | 0.11±0.01 | 0.19±0.02 |
| 1-Nonanol | $C_9H_{20}O$ | 143-08-8 | 20.4588 | 1171/1173 | 806/819 | 0.68±0.09 | 0.87±0.09 | 0.79±0.08 | 0.58±0.05 | 1.19±0.03 | 1.04±0.10 | 1.09±0.19 | 1.15±0.18 | 1.03±0.12 | 0.92±0.03 |
| Linalool oxide III | $C_{10}H_{18}O_2$ | 14009-71-3 | 20.4595 | 1172/1171 | 895/905 | 2.25±0.46 | 2.41±0.14 | 2.23±0.33 | 3.07±0.32 | 5.34±0.44 | 15.76±20.43 | 3.76±2.89 | 5.63±0.19 | 4.02±0.21 | 4.24±0.87 |
| Linalool oxide Ⅳ | $C_{10}H_{18}O_2$ | 14049-11-7 | 20.6588 | 1177/1173 | 893/897 | 1.63±0.25 | 1.15±0.19 | 1.01±0.12 | 1.09±0.11 | 2.09±0.17 | 3.02±0.19 | 2.76±0.49 | 3.17±0.12 | 4.65±0.35 | 5.35±1.34 |
| Terpinen-4-ol | $C_{10}H_{18}O$ | 562-74-3 | 20.7926 | 1181/1177 | 901/908 | 132.57±9.52 | 101.94±13.28 | 84.78±4.67 | 82.46±9.47 | 178.87±15.8 | 160.09±9.16 | 190.26±21.12 | 191.8±29.56 | 195.56±8.68 | 208.49±15.98 |
| *p*-Cymen-8-ol | $C_{10}H_{14}O$ | 1197-01-9 | 21.0577 | 1188/1183 | 854/873 | 0.47±0.09 | 0.50±0.05 | 0.39±0.03 | 0.31±0.04 | 0.77±0.05 | 0.66±0.01 | 0.60±0.10 | 0.76±0.02 | 0.72±0.04 | 0.82±0.07 |
| $\alpha$-Terpineol | $C_{10}H_{18}O$ | 98-55-5 | 21.2577 | 1193/1189 | 907/917 | 7.98±0.51 | 7.55±1.04 | 6.47±0.48 | 6.61±0.69 | 12.32±1.12 | 10.65±0.39 | 10.97±1.05 | 11.37±0.21 | 9.8±0.34 | 9.51±0.83 |
| (*Z*)-Piperitol | $C_{10}H_{18}O$ | 16721-38-3 | 21.4577 | 1199/1195 | 857/901 | 0.43±0.10 | 0.29±0.05 | 0.3±0.07 | 0.24±0.02 | 0.44±0.04 | 0.37±0.02 | 0.38±0.08 | 0.40±0.02 | 0.34±0.02 | 0.26±0.01 |
| Myrtenol | $C_{10}H_{16}O$ | 515-00-4 | 21.5261 | 1201/1195 | 862/867 | 0.93±0.16 | 1.00±0.06 | 0.8±0.15 | 0.72±0.08 | 2.16±0.17 | 2.21±0.09 | 2.43±0.29 | 2.3±0.29 | 2.49±0.09 | 2.27±0.13 |
| (*E*)-Isopiperitenol | $C_{10}H_{16}O$ | 74410-00-7 | 21.6577 | 1205/1210 | 893/895 | 1.58±0.04 | 1.79±0.26 | 1.53±0.15 | 1.76±0.18 | 4.35±0.36 | 4.19±0.12 | 4.65±0.59 | 5.17±0.18 | 4.65±0.14 | 4.23±0.27 |
| (*E*)-Piperitol | $C_{10}H_{18}O$ | 16721-39-4 | 21.8577 | 1210/1208 | 808/834 | 2.16±0.11 | 2.18±0.34 | 1.81±0.08 | 1.77±0.19 | 2.97±2.16 | 3.88±0.27 | 3.5±0.48 | 3.76±0.12 | 3.29±0.29 | 3.17±0.24 |
| (Z)-Isopiperitenol | $C_{10}H_{16}O$ | 96555-02-1 | 22.2596 | 1222/1228 | 895/896 | 1.50±0.20 | 1.50±0.24 | 1.43±0.07 | 1.80±0.19 | 4.37±0.29 | 4.79±0.09 | 5.75±0.79 | 6.69±0.26 | 5.55±0.20 | 4.06±0.22 |
| Nerol | $C_{10}H_{18}O$ | 106-25-2 | 22.5243 | 1229/1228 | 899/899 | 29.18±4.28 | 31.57±4.98 | 20.82±2.56 | 21.47±3.4 | 86.79±2.23 | 89.48±7.78 | 102.42±18.99 | 109.16±4.85 | 94.57±4.17 | 76.17±8.77 |
| Citronellol | $C_{10}H_{20}O$ | 106-22-9 | 22.5243 | 1229/1228 | 851/867 | 0.32±0.01 | 0.32±0.04 | 0.29±0.01 | 0.25±0.03 | 0.58±0.06 | 0.70±0.06 | 0.60±0.00 | 0.89±0.03 | 0.72±0.07 | 0.31±0.06 |
| (*Z*)-*p*-1(6),8-menthadien-2-ol | $C_{10}H_{16}O$ | 1197-06-4 | 22.6618 | 1233/1229 | 839/839 | RD | RD | RD | RD | RD | 0.57±0.04 | 0.55±0.14 | 0.67±0.10 | 0.64±0.08 | 0.63±0.10 |
| Isogeraniol | $C_{10}H_{18}O$ | 5944-20-7 | 23.0578 | 1245/1240 | 796/827 | 0.06±0.02 | 0.06±0.01 | 0.05±0.00 | 0.05±0.01 | 0.13±0.02 | 0.14±0.01 | 0.12±0.02 | 0.10±0.02 | 0.08±0.02 | 0.06±0.01 |
| Geraniol | $C_{10}H_{18}O$ | 106-24-1 | 23.3909 | 1254/1255 | 877/877 | 347.44±12.79 | 398.55±23.99 | 298.76±23.06 | 326.05±34.84 | 725.63±77.57 | 683.87±46.30 | 701.71±62.75 | 721.6±125.24 | 689.93±7.66 | 713.14±69.01 |
| *p*-Mentha-1,8-dien-7- | $C_{10}H_{16}O$ | 536-59-4 | 24.7244 | 1303/1297 | 766/841 | 0.38±0.03 | 0.78±0.15 | 0.47±0.14 | 0.38±0.04 | 1.23±0.14 | 1.04±0.10 | 0.91±0.16 | 0.86±0.04 | 0.71±0.05 | 0.54±0.06 |

| | | | | | | | | | | | | | | | |
|---|---|---|---|---|---|---|---|---|---|---|---|---|---|---|---|
| ol | | | | | | | | | | | | | | | |
| *p*-Menth-1-en-9-ol | C10H18O | 18479-68-0 | 24.9909 | 1301/1295 | 891/895 | 0.07±0.00 | 0.1±0.01 | 0.08±0.01 | 0.07±0.01 | 0.16±0.03 | 0.15±0.02 | 0.09±0.02 | 0.14±0.01 | 0.11±0.02 | 0.12±0.02 |
| Nerolidol | C15H26O | 40716-66-3 | 33.3242 | 1566/1564 | 915/919 | 7.05±0.78 | 1.40±0.06 | 0.75±0.07 | 0.84±0.11 | 2.48±0.28 | 2.30±0.06 | 2.64±0.41 | 3.28±0.37 | 2.66±0.18 | 2.75±0.4 |
| δ-Cadinol | C15H26O | 19435-97-3 | 35.7258 | 1655/1645 | 850/857 | 0.19±0.04 | 0.14±0.02 | 0.08±0.02 | 0.09±0.01 | 0.24±0.02 | 0.15±0.01 | 0.16±0.02 | 0.21±0.01 | 0.16±0.02 | 0.21±0.04 |
| **Esters** | | | | | | | | | | | | | | | |
| Methyl-hex-2-enoate | C7H12O2 | 13894-63-8 | 12.8575 | 966/966 | 893/899 | 0.05±0.01 | 0.11±0.00 | 0.15±0.01 | 0.39±0.05 | 0.76±0.05 | 0.77±0.08 | 0.79±0.01 | 0.62±0.04 | 0.52±0.08 | 0.14±0.03 |
| Ethyl caproate | C8H16O2 | 123-66-0 | 14.1245 | 1000/999 | 868/875 | 2.03±0.09 | 1.88±0.13 | 1.77±0.04 | 1.26±0.13 | 2.10±0.10 | 1.72±0.08 | 1.5±0.07 | 1.08±0.81 | 1.22±0.09 | 1.28±0.08 |
| (*E*)-3-Hexenyl acetate | C8H14O2 | 3681-82-1 | 14.3928 | 1008/1005 | 872/912 | 13.55±1.18 | 13.66±0.53 | 11.14±0.62 | 6.81±0.72 | 6.41±0.95 | 3.11±0.21 | 1.48±0.07 | 0.71±0.11 | RD | RD |
| Hexyl ethanoate | C8H16O2 | 142-92-7 | 14.6576 | 1015/1011 | 762/812 | 0.37±0.04 | 0.87±0.05 | 0.69±0.04 | 0.52±0.06 | 0.91±0.03 | 0.45±0.06 | 0.30±0.03 | 0.21±0.05 | 0.14±0.03 | 0.09±0.01 |
| (*E*)-2-Hexenyl acetate | C8H16O2 | 2497-18-9 | 14.7241 | 1017/1015 | 870/923 | 0.79±0.10 | 2.13±0.21 | 1.30±0.06 | 0.68±0.07 | 1.13±0.05 | 0.46±0.15 | RD | RD | RD | RD |
| 5-Ethyldihydro-2(3h)-furanone | C6H10O2 | 695-06-7 | 16.26 | 1057/1056 | 782/896 | RD | RD | 0.30±0.41 | 0.07±0.01 | 0.11±0.01 | 0.05±0.01 | 0.12±0.00 | 0.06±0.01 | 0.06±0.02 | 0.12±0.01 |
| Methylbenzoate | C8H8O2 | 93-58-3 | 17.7242 | 1097/1094 | 900/920 | 0.31±0.09 | 0.83±0.25 | 0.58±0.10 | 0.10±0.02 | 0.60±0.20 | 0.59±0.12 | 0.58±0.15 | 0.23±0.06 | 0.81±0.08 | 0.24±0.09 |
| Benzyl acetate | C9H10O2 | 140-11-4 | 20.2577 | 1166/1164 | 900/928 | 1.14±0.09 | 0.87±0.16 | 0.86±0.15 | 0.24±0.06 | 1.57±0.56 | 2.48±0.67 | 1.75±0.91 | 2.79±0.17 | 4.24±0.45 | 1.16±0.17 |
| (*E*)-Hex-3-enyl butyrate | C10H18O2 | 53398-84-8 | 21.0577 | 1188/1185 | 918/927 | 17.19±1.29 | 14.81±1.54 | 14.61±0.74 | 12.47±1.29 | 17.7±1.55 | 13.62±0.84 | 10.91±1.00 | 8.58±0.21 | 5.29±0.30 | 3.58±0.37 |
| Hexyl butanoate | C10H20O2 | 2639-63-6 | 21.2577 | 1193/1192 | 854/876 | 0.73±0.11 | 0.98±0.22 | 1.46±0.10 | 2.11±0.36 | 8.86±0.59 | 7.05±0.68 | 5.14±0.58 | 3.99±0.14 | 2.38±0.18 | 1.58±0.24 |
| (*E*)-2-Hexenyl butyrate | C10H18O2 | 53398-83-7 | 21.3243 | 1188/1195 | 856/875 | 1.00±0.19 | 3.48±0.25 | 2.57±0.16 | 2.50±0.26 | 7.32±0.38 | 5.59±0.31 | 4.06±0.37 | 2.83±0.28 | 1.72±0.07 | 1.06±0.09 |
| Methyl salicylate | C8H8O3 | 119-36-8 | 21.3908 | 1197/1192 | 887/889 | 282.91±25.48 | 321.58±23.88 | 310.66±22.34 | 309.89±34.06 | 691±52.17 | 636.1±29.99 | 639.21±68.86 | 646.75±15.5 | 573.04±20.93 | 518.61±26.62 |
| Ethyl nonanoate | C10H20O2 | 106-32-1 | 21.3908 | 1197/1196 | 822/868 | 1.09±0.11 | 0.74±0.09 | 0.77±0.11 | 0.68±0.08 | 0.23±0.12 | 0.37±0.01 | 0.26±0.04 | 1.08±1.3 | 0.30±0.00 | 0.35±0.03 |
| (*Z*)-3-Hexenyl-α- | C11H20O | 53398-85-9 | 22.6578 | 1233/1234 | 912/924 | 7.15±0.94 | 6.56±0.61 | 7.75±0.47 | 7.02±0.84 | 11.23±1.18 | 9.67±0.78 | 8.42±0.49 | 8.06±0.18 | 6.12±0.24 | 5.06±0.41 |

| | | | | | | | | | | | | | | | |
|---|---|---|---|---|---|---|---|---|---|---|---|---|---|---|---|
| methylbutyrate | 2 | | | | | | | | | | | | | | |
| (*E*)-2-Hexenyl isovalerate | C11H20O2 | 68698-59-9 | 22.8578 | 1239/1245 | 839/895 | 1.24±0.22 | 2.71±0.30 | 2.48±0.22 | 2.79±0.49 | 5.19±0.22 | 4.81±0.22 | 4.18±0.38 | 3.72±0.21 | 2.92±0.05 | 2.31±0.15 |
| Hexyl 2-methylbutanoate | C11H22O2 | 10032-15-2 | 22.8578 | 1239/1236 | 885/891 | 0.07±0.00 | 0.12±0.02 | 0.24±0.03 | 0.31±0.03 | 0.51±0.15 | 0.93±0.06 | 0.67±0.53 | 0.87±0.02 | 0.67±0.02 | 0.65±0.05 |
| Phenethyl acetate | C10H12O2 | 103-45-7 | 23.5909 | 1261/1258 | 758/914 | RD | 0.11±0.02 | 0.12±0.01 | 0.11±0.01 | 0.29±0.09 | 0.25±0.02 | 0.24±0.01 | 0.21±0.05 | 0.06±0.01 | RD |
| Ethyl salicylate | C9H10O3 | 118-61-6 | 24.06 | 1274/1270 | 713/889 | 0.11±0.01 | 0.26±0.12 | 0.18±0.01 | 0.21±0.02 | 0.85±0.11 | 0.89±0.12 | 1.03±0.16 | 1.01±0.03 | 0.93±0.03 | RD |
| Prenyl hexanoate | C11H20O2 | 76649-22-4 | 24.5931 | 1289/1284 | 782/816 | RD | RD | RD | RD | 0.69±0.04 | 0.66±0.12 | 0.58±0.02 | 0.56±0.10 | 0.38±0.05 | 0.23±0.04 |
| Ethyl pelargonate | C11H22O2 | 123-29-5 | 24.8575 | 1296/1295 | 824/847 | 0.32±0.04 | 0.47±0.17 | 0.14±0.00 | 0.14±0.02 | 0.18±0.02 | 0.19±0.03 | 0.17±0.02 | 0.18±0.02 | 0.16±0.02 | 0.09±0.02 |
| Methyl geranate | C11H18O2 | 1189-09-9 | 25.7928 | 1325/1322 | 867/885 | 53.4±4.55 | 30.29±1.76 | 22.42±1.91 | 35.73±4.82 | 112.31±10.48 | 127.28±2.66 | 147.72±14.63 | 173.43±6.89 | 162.14±6.71 | 149.15±12.51 |
| 3-Hydroxy-2,2,4-trimethylpentyl isobutyrate | C12H24O3 | 77-68-9 | 27.5244 | 1378/1374 | 735/818 | 0.06±0.01 | 0.05±0.01 | 0.04±0.00 | 0.06±0.01 | 0.07±0.01 | 0.06±0.01 | 0.11±0.01 | 0.10±0.04 | 0.07±0.01 | 0.07±0.02 |
| (Z)-3-Hexenyl hexanoate | C12H22O2 | 31501-11-8 | 27.6575 | 1382/1380 | 873/889 | 8.52±0.35 | 8.56±0.88 | 9.81±0.58 | 9.26±0.96 | 21.33±1.72 | 17.8±1.07 | 16.07±1.54 | 15.49±0.53 | 11.95±0.48 | 10.45±1.04 |
| Hexanoic acid, hexyl ester | C12H24O2 | 6378-65-0 | 27.7932 | 1388/1384 | 881/887 | RD | RD | RD | RD | 3.92±0.36 | 3.48±0.13 | 3.25±0.32 | 2.32±0.55 | 2.62±0.02 | 2.39±0.25 |
| (Z)-3-Hexenyl (Z)-3-hexenoate | C12H24O3 | 61444-38-0 | 27.8576 | 1388/1389 | 855/872 | 1.40±0.07 | 1.84±0.16 | 1.85±0.17 | 1.66±0.17 | 4.05±0.19 | 3.05±0.07 | 2.50±0.27 | RD | RD | RD |
| (*E*)-2-Hexenyl Hexanoate | C12H22O2 | 53398-86-0 | 27.9245 | 1390/1391 | 838/851 | 0.56±0.04 | 1.70±0.17 | 1.84±0.10 | 2.16±0.22 | 9.89±0.75 | 8.32±0.41 | 7.35±0.66 | 9.18±0.31 | 6.54±0.32 | 5.21±0.49 |
| *β*-Phenylethyl | C12H16O | 103-52-6 | 29.6602 | 1446/1444 | 860/881 | RD | RD | RD | 0.03±0.00 | 0.10±0.01 | 0.09±0.01 | 0.09±0.02 | 0.09±0.00 | 0.08±0.00 | 0.05±0.00 |

| | | | | | | | | | | | | | | | |
|---|---|---|---|---|---|---|---|---|---|---|---|---|---|---|---|
| butyrate | 2 | | | | | | | | | | | | | | |
| Dimethyl phthalate | C10H10O4 | 131-11-3 | 30.1242 | 1459/1454 | 867/882 | 0.21±0.02 | 0.13±0.01 | 0.11±0.01 | 0.11±0.03 | 0.12±0.02 | 0.27±0.05 | 0.23±0.05 | 0.19±0.02 | 0.22±0.01 | 0.13±0.01 |
| (*Z*)-3-Hexenyl benzoate | C13H16O2 | 25152-85-6 | 33.5908 | 1576/1570 | 938/946 | 0.85±0.15 | 0.37±0.04 | 0.45±0.04 | 0.50±0.06 | 1.20±0.20 | 1.13±0.03 | 1.10±0.15 | 1.24±0.09 | 1.29±0.09 | 0.89±0.03 |
| Benzoic acid, hexyl ester | C13H18O2 | 6789-88-4 | 33.7934 | 1583/1580 | 835/861 | RD | RD | RD | 0.03±0.01 | 0.09±0.01 | 0.07±0.01 | 0.11±0.02 | 0.15±0.02 | 0.13±0.06 | 0.11±0.03 |
| (*E*)-2-Hexenyl benzoate | C13H16O2 | 76841-70-8 | 33.9935 | 1590/1588 | 843/873 | RD | RD | RD | 0.04±0.01 | 0.24±0.03 | 0.21±0.02 | 0.22±0.03 | 0.26±0.01 | 0.24±0.03 | 0.19±0.04 |
| | | | | | | | **Ketones** | | | | | | | | |
| 2-Heptanone | C7H14O | 110-43-0 | 10.1909 | 892/891 | 793/819 | 0.27±0.02 | 0.33±0.03 | 0.45±0.07 | 0.32±0.03 | 0.31±0.04 | 0.24±0.06 | 0.26±0.00 | 0.13±0.01 | 0.14±0.02 | 0.12±0.02 |
| 6-Methyl-5-hepten-2-one | C8H14O | 110-93-0 | 13.6575 | 988/986 | 885/897 | RD | RD | RD | RD | RD | RD | RD | 1.12±0.48 | 1.65±0.60 | 0.78±0.34 |
| 3-Octanone | C8H16O | 106-68-3 | 13.6575 | 988/986 | 819/896 | 0.10±0.01 | 0.09±0.00 | 0.09±0.01 | 0.08±0.00 | 0.11±0.00 | 0.12±0.01 | 0.12±0.00 | 0.08±0.01 | 0.07±0.00 | 0.07±0.00 |
| 3-Octen-2-one | C8H14O | 1669-44-9 | 15.5934 | 1040/1040 | 842/897 | 0.70±0.10 | 0.79±0.10 | 0.77±0.05 | 0.6±0.07 | RD | RD | RD | RD | RD | RD |
| 3-Methyl-2-cyclohexen-1-one | C7H10O | 1193-18-6 | 16.4576 | 1063/1075 | 844/864 | 0.15±0.02 | 0.16±0.01 | 0.15±0.02 | 0.07±0.01 | 0.17±0.02 | 0.26±0.06 | 0.19±0.03 | 0.17±0.02 | 0.16±0.02 | 0.2±0.02 |
| Acetophenone | C8H8O | 98-86-2 | 16.7242 | 1071/1066 | 848/856 | 0.08±0.02 | 0.35±0.27 | 0.58±0.07 | 0.29±0.11 | 1.23±0.14 | 0.52±0.26 | 0.92±0.12 | 0.97±0.30 | 0.86±0.05 | 0.94±0.13 |
| (*E*, *E*)-3,5-Octadien-2-one | C8H12O | 30086-02-3 | 16.7911 | 1072/1073 | 853/864 | 4.57±0.37 | 5.28±0.51 | 5.02±0.15 | 4.10±0.43 | 2.64±0.16 | 2.79±0.29 | 2.27±0.05 | 2.32±0.31 | 2.29±0.29 | 3.35±0.29 |
| 3,5-Octadien-2-one | C8H12O | 38284-27-4 | 17.6576 | 1095/1091 | 845/871 | 1.09±0.15 | 0.93±0.71 | 1.37±0.14 | 1.14±0.12 | 0.49±0.04 | 0.55±0.04 | 0.35±0.06 | 0.57±0.03 | 0.39±0.02 | 0.66±0.04 |
| 5-Ethyl-6-methyl-3E-hepten-2-one | C10H18O | 57283-79-1 | 19.5908 | 1148/1144 | 837/859 | 0.85±0.08 | 1.10±0.06 | 1.24±0.09 | 1.10±0.12 | 0.47±0.05 | 0.42±0.02 | 0.30±0.04 | 0.30±0.01 | 0.30±0.02 | 0.74±0.01 |
| 5-Isopropylbicyclo [3.1.0]hexan-2-one | C9H14O | 513-20-2 | 20.1257 | 1163/1156 | 795/844 | 0.04±0.01 | 0.05±0.01 | 0.04±0.01 | 0.06±0.01 | 0.06±0.00 | 0.06±0.01 | 0.07±0.01 | 0.06±0.01 | 0.05±0.01 | 0.06±0.01 |
| Carvone | C10H14O | 2244-16-8 | 23.1909 | 1249/1246 | 857/906 | 0.05±0.00 | 0.05±0.01 | 0.04±0.01 | 0.07±0.01 | 0.42±0.04 | 0.37±0.07 | 0.35±0.10 | 0.34±0.06 | 0.34±0.08 | 0.32±0.03 |

| | | | | | | | | | | | | | | | |
|---|---|---|---|---|---|---|---|---|---|---|---|---|---|---|---|
| Isopiperitenone | C10H14O | 529-01-1 | 24.1273 | 1276/1272 | 832/856 | RD | 0.06±0.01 | 0.10±0.02 | 0.16±0.02 | 0.65±0.09 | 0.65±0.19 | 0.51±0.08 | 0.77±0.03 | 0.68±0.06 | 0.62±0.04 |
| 2-Undecanone | C11H22O | 112-12-9 | 24.7909 | 1295/1294 | 720/832 | 0.11±0.01 | 0.14±0.03 | 0.11±0.01 | 0.10±0.01 | 0.18±0.01 | 0.13±0.01 | 0.15±0.01 | 0.17±0.04 | 0.16±0.02 | 0.14±0.01 |
| Damascenone | C13H18O | 23726-93-4 | 27.9287 | 1391/1386 | 724/843 | RD | RD | RD | RD | RD | RD | RD | 1.39±0.33 | RD | 0.69±0.13 |
| (*Z*)-Jasmone | C11H16O | 488-10-8 | 28.3245 | 1403/1395 | 889/894 | 305.67±23.96 | 180.98±23.2 | 134.02±5.98 | 124.33±13.95 | 224.85±19.34 | 178.04±11.13 | 187.84±27.7 | 201.91±7.14 | 172.46±8.51 | 170.02±14.31 |
| *α*-Ionone | C13H20O | 127-41-3 | 29.2576 | 1433/1426 | 880/880 | 3.03±0.28 | 4.01±0.31 | 3.35±0.45 | 3.11±0.35 | 2.83±0.23 | 2.47±0.13 | 2.86±0.50 | 3.57±0.28 | 3.09±0.32 | 3.10±0.31 |
| Geranylacetone | C13H22O | 3796-70-1 | 29.9922 | 1456/1453 | 879/886 | 0.90±0.050 | 1.10±0.09 | 0.81±0.13 | 0.79±0.21 | 1.23±0.25 | 1.34±0.03 | 1.28±0.16 | 1.50±0.12 | 1.34±0.13 | 1.22±0.20 |
| (*E*)-*β*-Ionone | C13H20O | 79-77-6 | 31.126 | 1492/1486 | 890/890 | 20.57±1.22 | 31.64±3.05 | 27.67±4.13 | 22.58±2.48 | 22.91±2.26 | 21.30±1.15 | 26.66±4.84 | 33.34±1.53 | 30.03±2.41 | 31.17±3.64 |
| Alkenes | | | | | | | | | | | | | | | |
| 1,2,5,5-Tetramethyl-1,3-cyclopentadiene | C9H14 | 4249-12-1 | 8.5934 | 842/840 | 851/862 | RD | RD | RD | 0.02±0.00 | 0.04±0.00 | 0.04±0.01 | 0.05±0.00 | 0.10±0.00 | 0.07±0.01 | 0.11±0.01 |
| Styrene | C8H8 | 100-42-5 | 10.1909 | 892/893 | 820/914 | 0.15±0.01 | 0.12±0.01 | 0.11±0.01 | 0.07±0.01 | 0.05±0.01 | 0.05±0.01 | 0.06±0.01 | 0.08±0.02 | 0.04±0.01 | 0.06±0.01 |
| *α*-Thujene | C10H16 | 2867-05-2 | 11.4593 | 927/929 | 822/851 | 0.32±0.04 | 0.26±0.02 | 0.21±0.01 | 0.17±0.04 | 0.23±0.01 | 0.16±0.03 | 0.15±0.02 | 0.16±0.02 | 0.11±0.02 | 0.10±0.03 |
| *α*-Pinene | C10H16 | 80-56-8 | 11.7244 | 935/937 | 877/886 | 0.11±0.01 | 0.06±0.00 | 0.04±0.00 | 0.03±0.00 | 0.05±0.00 | 0.04±0.00 | 0.05±0.00 | 0.05±0.00 | 0.03±0.00 | RD |
| Dehydrosabinene | C10H14 | 36262-09-6 | 12.1262 | 946/956 | 777/830 | 0.12±0.01 | 0.13±0.01 | 0.11±0.00 | 0.10±0.01 | 0.10±0.01 | 0.09±0.01 | 0.09±0.00 | 0.14±0.01 | 0.07±0.01 | 0.10±0.01 |
| Sabinene | C10H16 | 3387-41-5 | 13.1928 | 975/974 | 915/918 | 0.45±0.01 | 0.34±0.02 | 0.30±0.07 | 0.17±0.03 | 0.15±0.02 | 0.11±0.01 | 0.09±0.00 | 0.07±0.02 | 0.05±0.01 | 0.03±0.01 |
| *β*-Myrcene | C10H16 | 123-35-3 | 13.791 | 991/991 | 904/919 | 0.36±0.09 | 0.20±0.03 | 0.04±0.02 | 0.07±0.02 | 0.55±0.19 | 0.56±0.06 | 0.58±0.04 | 0.65±0.08 | 0.49±0.08 | 0.62±0.09 |
| *α*-Phellandrene | C10H16 | 99-83-2 | 14.3245 | 1006/1005 | 907/918 | 0.94±0.19 | 0.80±0.12 | 0.50±0.03 | 0.38±0.06 | 1.41±0.43 | 0.9±0.02 | 1.03±0.10 | 0.91±0.03 | 0.77±0.16 | 0.97±0.06 |
| *α*-Terpinene | C10H16 | 99-86-5 | 14.791 | 1018/1017 | 922/932 | 8.37±0.53 | 6.5±1.06 | 5.11±0.23 | 4.54±0.66 | 8.51±0.85 | 7.34±0.36 | 8.23±0.68 | 8.24±0.27 | 6.81±0.5 | 7.97±0.78 |
| D-Limonene | C10H16 | 5989-27-5 | 15.2594 | 1031/1031 | 857/873 | 2.35±0.17 | 1.38±0.14 | 1.15±0.06 | 1.28±0.24 | 2.73±0.36 | 2.79±0.47 | 3.62±1.01 | 4.94±1.14 | 4.45±0.69 | 3.46±0.28 |
| *β*-Phellandrene | C10H16 | 555-10-2 | 15.3241 | 1032/1031 | 890/923 | 1.06±0.06 | 0.73±0.07 | 0.58±0.03 | 0.91±0.68 | 1.25±0.21 | 1.30±0.26 | 1.24±0.15 | 2.82±2.15 | 1.01±0.06 | 1.56±0.16 |
| *β*-Ocimene | C10H16 | 13877-91-3 | 15.591 | 1040/1037 | 896/903 | 0.40±0.06 | 0.32±0.04 | 0.25±0.03 | 0.21±0.04 | 0.42±0.05 | 0.41±0.05 | 0.44±0.01 | 0.48±0.08 | 0.45±0.03 | 0.50±0.02 |
| (*E*)-β-Ocimene | C10H16 | 3779-61-1 | 15.9911 | 1050/1049 | 897/900 | 0.91±0.07 | 0.62±0.06 | 0.47±0.02 | 0.58±0.10 | 1.03±0.13 | 0.96±0.09 | 1.01±0.02 | 0.93±0.05 | 0.76±0.07 | 1.10±0.12 |
| γ-Terpinene | C10H16 | 99-85-4 | 16.3911 | 1061/1060 | 912/912 | 13.33±0.59 | 10.79±1.79 | 8.24±0.44 | 7.34±1.00 | 14.04±1.22 | 11.54±0.54 | 12.92±0.90 | 12.61±0.32 | 10.63±0.56 | 11.7±0.92 |
| Isoterpinene | C10H16 | 586-62-9 | 17.5242 | 1091/1088 | 913/917 | 2.66±0.06 | 2.00±0.24 | 1.49±0.10 | 1.32±0.18 | 2.53±0.32 | 2.31±0.11 | 2.76±0.02 | 2.81±0.29 | 2.46±0.24 | 2.61±0.11 |
| 1,3,8-p-Menthatriene | C10H14 | 18368-95-1 | 18.3911 | 1115/1119 | 865/876 | 0.74±0.08 | 0.7±0.06 | 0.50±0.03 | 0.39±0.06 | 0.64±0.05 | 0.57±0.07 | 0.57±0.04 | 0.65±0.03 | 0.56±0.02 | 0.91±0.07 |
| (*E*)-4,8- | C11H18 | 19945-61-0 | 18.5242 | 1118/1116 | 857/874 | 2.69±0.08 | 0.88±0.08 | 0.39±0.02 | 0.35±0.04 | 0.73±0.04 | 0.52±0.07 | 0.49±0.03 | 0.47±0.01 | 0.31±0.04 | 0.50±0.08 |

| | | | | | | | | | | | | | | | |
|---|---|---|---|---|---|---|---|---|---|---|---|---|---|---|---|
| Dimethylnona-1,3,7-triene | | | | | | | | | | | | | | | |
| (*E*, *E*)-2,6-Dimethyl-1,3,5,7-octatetraene | C10H14 | 460-01-5 | 19.0599 | 1133/1131 | 758/912 | RD | RD | RD | RD | 0.26±0.02 | 0.25±0.02 | 0.23±0.02 | 0.21±0.02 | 0.20±0.01 | 0.47±0.06 |
| Safrole | C10H10O2 | 94-59-7 | 24.7244 | 1293/1287 | 798/811 | 0.12±0.01 | 0.19±0.03 | 0.14±0.01 | 0.10±0.01 | 0.18±0.04 | 0.19±0.01 | 0.19±0.02 | 0.2±0.03 | 0.18±0.02 | 0.16±0.00 |
| 1-Ethylidene-1H-indene | C11H10 | 2471-83-2 | 25.5244 | 1318/1315 | 865/885 | 0.35±0.04 | 0.29±0.04 | 0.24±0.02 | 0.15±0.02 | 0.23±0.03 | 0.20±0.04 | 0.18±0.02 | 0.21±0.02 | 0.18±0.01 | 0.14±0.01 |
| *α*-Cubebene | C15H24 | 17699-14-8 | 26.791 | 1355/1351 | 765/841 | 0.08±0.01 | 0.06±0.01 | 0.05±0.00 | 0.05±0.01 | 0.11±0.02 | 0.08±0.01 | 0.08±0.00 | 0.10±0.03 | 0.06±0.02 | 0.05±0.01 |
| 1-Tetradecene | C14H28 | 1120-36-1 | 27.991 | 1392/1392 | 785/834 | 0.11±0.01 | 0.07±0.00 | 0.08±0.02 | 0.12±0.01 | 0.18±0.05 | 0.15±0.02 | 0.13±0.02 | 0.13±0.01 | 0.12±0.02 | 0.1±0.03 |
| Caryophyllene | C15H24 | 87-44-5 | 28.7241 | 1419/1419 | 843/846 | 0.08±0.02 | 0.14±0.03 | 0.11±0.01 | 0.10±0.01 | 0.21±0.01 | 0.12±0.01 | 0.14±0.02 | 0.15±0.01 | 0.11±0.01 | 0.15±0.01 |
| Longifolene | C15H24 | 475-20-7 | 28.7259 | 1415/1406 | 783/817 | 0.07±0.01 | 0.05±0.01 | 0.05±0.00 | 0.05±0.01 | 0.05±0.01 | 0.06±0.01 | 0.06±0.01 | 0.05±0.01 | 0.03±0.00 | RD |
| Cedrene | C15H24 | 11028-42-5 | 28.9241 | 1422/1422 | 850/859 | 0.11±0.03 | 0.11±0.00 | 0.08±0.00 | 0.07±0.01 | 0.14±0.02 | 0.10±0.00 | 0.10±0.01 | 0.1±0.01 | 0.09±0.00 | 0.08±0.01 |
| (*E*)-*β*-Farnesene | C15H24 | 18794-84-8 | 30.1242 | 1460/1457 | 758/815 | 0.21±0.03 | 0.10±0.01 | 0.05±0.02 | 0.06±0.01 | 0.1±0.03 | 0.09±0.00 | 0.09±0.00 | 0.08±0.01 | 0.07±0.00 | 0.09±0.01 |
| *α*-Muurolene | C15H24 | 10208-80-7 | 31.5911 | 1507/1499 | 832/836 | 0.08±0.00 | 0.05±0.00 | 0.04±0.00 | 0.04±0.01 | 0.09±0.01 | 0.06±0.01 | 0.07±0.01 | 0.07±0 | 0.05±0.01 | 0.08±0.01 |
| *α*-Farnesene | C15H24 | 502-61-4 | 31.6576 | 1510/1508 | 807/822 | 0.21±0.01 | 0.08±0.02 | 0.04±0.01 | 0.02±0.00 | 0.05±0.02 | 0.05±0.00 | 0.05±0.01 | 0.06±0.01 | 0.05±0.01 | 0.05±0.00 |
| (*E*)-Calamenene | C15H22 | 73209-42-4 | 32.2577 | 1530/1529 | 837/880 | 0.13±0.02 | 0.11±0.01 | 0.08±0.01 | 0.08±0.01 | 0.19±0.01 | 0.13±0.01 | 0.15±0.02 | 0.17±0.00 | 0.14±0.00 | 0.21±0.02 |
| δ-Cadinene | C15H24 | 483-76-1 | 32.2577 | 1530/1524 | 901/903 | 0.47±0.02 | 0.43±0.05 | 0.26±0.01 | 0.30±0.04 | 0.81±0.03 | 0.53±0.02 | 0.63±0.08 | 0.52±0.41 | 0.37±0.25 | 0.5±0.36 |
| *α*-Calacorene | C15H20 | 21391-99-1 | 32.9261 | 1552/1542 | 849/907 | RD | RD | RD | 0.02±0.00 | 0.08±0.00 | 0.06±0.01 | 0.08±0.02 | 0.09±0.01 | 0.09±0.01 | 0.13±0.01 |
| | | | | | | | Aromatic compounds | | | | | | | | |
| Toluene | C7H8 | 108-88-3 | 6.3908 | 761/763 | 805/865 | 0.21±0.05 | 0.11±0.01 | 0.11±0.00 | RD | RD | RD | RD | RD | RD | RD |
| *p*-Xylene | C8H10 | 106-42-3 | 9.4578 | 869/865 | 851/929 | 0.23±0.01 | 0.32±0.03 | 0.13±0.03 | 0.10±0.01 | RQ | RQ | RQ | RQ | RQ | RQ |
| *p*-Cymene | C10H14 | 99-87-6 | 15.1241 | 1027/1025 | 880/903 | 4.25±0.69 | 3.17±0.31 | 3.02±0.37 | 3.15±0.38 | 6.11±0.17 | 5.72±0.78 | 6.35±0.33 | 7.10±0.30 | 7.40±0.45 | 12.64±1.11 |
| 1-Methyl-4-(1-methylethenyl)-benzene | C10H12 | 1195-32-0 | 17.526 | 1092/1090 | 804/887 | 2.03±0.11 | 1.96±0.14 | 1.7±0.05 | 1.52±0.23 | 2.42±0.15 | 2.3±0.14 | 2.52±0.07 | 2.43±0.14 | 2.57±0.04 | 4.51±0.64 |

| | | | | | | | | | | | | | | | |
|---|---|---|---|---|---|---|---|---|---|---|---|---|---|---|---|
| Naphthalene | C10H8 | 91-20-3 | 21.0577 | 1188/1182 | 885/928 | 1.62±0.13 | 1.38±0.15 | 1.08±0.08 | 0.74±0.10 | 1.04±0.08 | 0.82±0.04 | 0.72±0.09 | 0.85±0.02 | 0.70±0.04 | 0.83±0.06 |
| 2-Methyl-naphthalene | C11H10 | 91-57-6 | 24.9244 | 1299/1297 | 880/906 | 0.79±0.09 | 0.82±0.10 | 0.59±0.06 | 0.30±0.04 | 0.51±0.09 | 0.43±0.06 | 0.36±0.03 | 0.43±0.06 | 0.36±0.01 | 0.24±0.05 |
| Biphenyl | C12H10 | 92-52-4 | 27.7244 | 1385/1381 | 813/877 | 0.19±0.04 | 0.18±0.01 | 0.13±0.01 | 0.07±0.01 | 0.11±0.02 | 0.09±0.01 | 0.07±0.01 | 0.09±0.00 | 0.08±0.01 | 0.05±0.00 |
| Fluorene | C13H10 | 86-73-7 | 33.9908 | 1590/1583 | 726/836 | 0.09±0.00 | 0.08±0.01 | 0.04±0.01 | 0.03±0.00 | 0.06±0.01 | 0.06±0.01 | 0.06±0.01 | 0.06±0.01 | 0.05±0.01 | 0.05±0.00 |
| Acids | | | | | | | | | | | | | | | |
| Nonanoic acid | C9H18O2 | 112-05-0 | 23.7266 | 1264/1273 | 748/843 | RD | RD | RD | RD | 0.05±0.01 | 0.08±0.00 | 0.07±0.01 | 0.09±0.01 | 0.13±0.02 | 0.07±0.02 |
| Geranic acid | C10H16O2 | 459-80-3 | 26.6575 | 1352/1355 | 887/893 | 0.1±0.01 | 0.09±0.02 | 0.08±0.00 | 0.21±0.02 | 5.12±0.45 | 2.97±3.90 | 10.9±2.23 | 12.94±1.89 | 11.72±1.03 | 12.58±0.96 |
| Alkanes | | | | | | | | | | | | | | | |
| Octane | C8H18 | 111-65-9 | 7.1908 | 797/800 | 828/898 | 0.06±0.01 | 0.05±0.00 | 0.04±0.00 | 0.03±0.00 | 0.05±0.00 | RD | RD | RD | RD | RD |
| 2,2,4,6,6-Pentamethyl-heptane | C12H26 | 13475-82-6 | 13.791 | 991/990 | 914/922 | 0.30±0.02 | 0.38±0.04 | 0.29±0.02 | 0.19±0.02 | 0.27±0.03 | 0.14±0.01 | 0.15±0.00 | 0.22±0.02 | 0.21±0.01 | 0.08±0.01 |
| Dodecane | C12H26 | 112-40-3 | 21.5243 | 1200/1200 | 706/870 | 0.23±0.05 | 0.19±0.02 | 0.30±0.08 | 0.12±0.04 | 0.21±0.03 | 0.19±0.03 | 0.17±0.03 | 0.23±0.02 | 0.26±0.06 | 0.23±0.10 |
| 2,6,11-trimethyl-Dodecane | C15H32 | 31295-56-4 | 24.3244 | 1281/1275 | 823/848 | 0.08±0.01 | 0.08±0.01 | 0.06±0.01 | 0.04±0.01 | 0.08±0.02 | 0.07±0.01 | 0.05±0.02 | 0.07±0.01 | 0.08±0.02 | 0.11±0.01 |
| Tridecane | C13H28 | 629-50-5 | 24.9935 | 1300/1300 | 838/879 | RD | 0.11±0.01 | 0.20±0.02 | 0.17±0.03 | 0.33±0.08 | 0.23±0.01 | 0.30±0.07 | 0.16±0.01 | 0.15±0.03 | 0.12±0.03 |
| 3-Methyl-tridecane | C14H30 | 6418-41-3 | 27.3244 | 1371/1371 | 837/870 | 0.12±0.01 | 0.10±0.01 | 0.08±0.01 | 0.09±0.01 | 0.16±0.02 | 0.12±0.02 | 0.12±0.01 | 0.11±0.02 | 0.11±0.02 | 0.11±0.01 |
| Tetradecane | C14H30 | 629-59-4 | 28.2576 | 1400/1400 | 896/908 | 0.55±0.06 | 0.39±0.03 | 0.36±0.04 | 0.33±0.05 | 0.54±0.07 | 0.46±0.04 | 0.54±0.04 | 0.49±0.1.00 | 0.44±0.06 | 0.41±0.02 |
| Octylcyclohexane | C14H28 | 1795-15-9 | 29.7929 | 1449/1448 | 836/846 | 0.11±0.01 | 0.08±0.01 | 0.07±0.00 | 0.06±0.01 | 0.11±0.01 | 0.09±0.02 | 0.11±0.00 | 0.09±0.00 | 0.08±0.00 | 0.08±0.01 |
| 3-Methyl-pentadecane | C16H34 | 2882-96-4 | 33.4577 | 1571/1570 | 830/850 | 0.29±0.05 | 0.17±0.01 | 0.10±0.01 | 0.1±0.01 | 0.17±0.02 | 0.16±0.02 | 0.17±0.02 | 0.17±0.02 | 0.17±0.03 | 0.11±0.01 |
| Hexadecane | C16H34 | 544-76-3 | 34.3243 | 1600/1600 | 887/902 | 0.49±0.06 | 0.34±0.02 | 0.25±0.10 | 0.21±0.03 | 0.39±0.11 | 0.29±0.02 | 0.37±0.03 | 0.38±0.02 | 0.25±0.06 | 0.33±0.04 |
| Heptadecane | C17H36 | 629-78-7 | 36.9243 | 1700/1700 | 856/871 | 0.19±0.03 | 0.22±0.02 | 0.12±0.02 | 0.08±0.01 | 0.11±0.08 | 0.16±0.01 | 0.17±0.01 | 0.17±0.01 | 0.13±0.04 | 0.11±0.01 |
| Oxygen heterocyclic compounds | | | | | | | | | | | | | | | |

| | | | | | | | | | | | | | | | |
|---|---|---|---|---|---|---|---|---|---|---|---|---|---|---|---|
| 2-n-Butyl furan | C8H12O | 4466-24-4 | 10.1909 | 892/893 | 827/839 | 0.35±0.04 | 0.27±0.01 | 0.29±0.08 | 0.27±0.06 | 0.16±0.06 | 0.12±0.01 | 0.1±0.02 | 0.19±0.06 | RD | RD |
| 2-Pentyl-furan | C9H14O | 3777-69-3 | 13.8575 | 993/993 | 889/895 | 1.71±0.02 | 1.73±0.29 | 1.96±0.21 | 2.35±0.27 | 1.92±0.25 | 1.61±0.3 | 1.6±0.34 | 1.66±0.12 | 2.09±0.16 | 3.5±0.32 |
| 1,8-Cineole | C10H18O | 470-82-6 | 15.391 | 1034/1032 | 901/903 | 2.19±0.13 | 1.00±0.12 | 0.74±0.04 | 0.58±0.10 | 0.55±0.06 | 0.37±0.02 | 0.31±0.03 | RD | RD | RD |
| 2,3-Dihydro-benzofuran | C8H8O | 496-16-2 | 22.1243 | 1218/1224 | 877/894 | 0.28±0.01 | 0.24±0.03 | 0.23±0.01 | 0.26±0.03 | 0.51±0.01 | 0.52±0.04 | 0.54±0.15 | 0.54±0.05 | 0.45±0.04 | 0.55±0.05 |
| Dibenzofuran | C12H8O | 132-64-9 | 31.9911 | 1522/1515 | 803/827 | 0.10±0.00 | 0.05±0.01 | 0.05±0.02 | RD | RD | RD | RD | RD | RD | RD |
| Other compounds | | | | | | | | | | | | | | | |
| Camphor | C10H16O | 464-48-2 | 19.6577 | 1150/1145 | 842/869 | 0.06±0.01 | 0.15±0.02 | 0.08±0.00 | 0.06±0.01 | 0.09±0.01 | 0.08±0.01 | 0.08±0.01 | 0.08±0.02 | 0.07±0.00 | 0.10±0.01 |
| Benzothiazole | C7H5NS | 95-16-9 | 22.5909 | 1230/1228 | 726/823 | 0.12±0.02 | 0.10±0.02 | 0.08±0.01 | 0.07±0.01 | 0.10±0.02 | 0.11±0.01 | 0.09±0.01 | 0.10±0.01 | 0.10±0.01 | 0.09±0.02 |
| Indole | C8H7N | 120-72-9 | 24.9244 | 1298/1295 | 874/879 | 24.16±1.77 | 15.52±2.08 | 7.57±0.35 | 5.34±0.58 | 7.80±0.58 | 4.57±0.80 | 2.84±0.16 | 1.75±0.07 | 0.52±0.03 | 0.34±0.01 |
| Dihydro-5-pentyl-2(3H)-furanone | C9H16O2 | 104-61-0 | 27.1267 | 1367/1365 | 868/876 | RD | RD | RD | 0.11±0.01 | 0.34±0.03 | 0.32±0.01 | 0.31±0.03 | 0.37±0.01 | 0.34±0.01 | 0.35±0.01 |
| 2,4-Di-tert-butylphenol | C14H22O | 96-76-4 | 31.7242 | 1512/1514 | 900/904 | 0.82±0.14 | 0.45±0.06 | 0.35±0.03 | 0.29±0.08 | 0.62±0.20 | 0.83±0.19 | 0.87±0.13 | 1.05±0.23 | 1.01±0.07 | 0.55±0.07 |

RD: represents cannot be detected; RQ: represents cannot be quantified. Retention index(RI) was calculated based on a series of n-alkanes.

Table S4. OAV of all volatiles in RCBT processing

| NO. | Compounds | OT (μg/L) | Description | FL | W1 | W2 | W3 | R | F1 | F2 | F3 | F4 | D |
|---|---|---|---|---|---|---|---|---|---|---|---|---|---|
| 1 | (*E*)-2-Pentenal | 1500 | Apple, green | 0.00±0.00 | 0.00±0.00 | 0.00±0.00 | 0.00±0.00 | 0.00±0.00 | 0.00±0.00 | 0.00±0.00 | 0.00±0.00 | 0.00±0.00 | 0.00±0.00 |
| 2 | Hexanal | 4.5 | Fat, sweet | 65.88±6.50 | 58.38±10.69 | 64.68±3.84 | 77.39±9.58 | 81.73±6.87 | 75.85±4.77 | 90.77±6.58 | 117.13±5.71 | 102.96±8.10 | 151.03±12.77 |
| 3 | (*E*)-2-Hexenal | / | Green apple, grass | | | | | | | | | | |
| 4 | (Z)-4-Heptenal | 0.06 | Baked potatoes, biscuit | 69.40±16.96 | 100.25±32.72 | 71.55±28.85 | 60.44±26.98 | 127.11±25.48 | 107.18±12.8 | 111.91±7.91 | 119.38±12.13 | 114±35.05 | 65.65±15.93 |
| 5 | Heptanal | 10 | Citrus, fat, green | 7.08±1.49 | 6.91±0.52 | 4.40±0.35 | 2.81±0.47 | 7.66±0.72 | 4.93±0.27 | 6.01±0.15 | 8.58±0.13 | 6.71±0.30 | 5.77±0.27 |
| 6 | (*E*, *E*)-2,4-Hexadienal | 60 | Citrus, fat, green | | | | | | | 0.02±0.01 | 0.09±0.02 | 0.08±0.03 | 0.04±0.00 |
| 7 | (*E*)-2-Heptenal | / | Fat, green | | | | | | | | | | |
| 8 | Benzaldehyde | 750.9 | Sweet, bitter almond | 0.04±0.01 | 0.03±0.01 | 0.02±0.00 | 0.01±0.00 | 0.01±0.01 | 0.01±0.00 | 0.01±0.00 | 0.02±0.00 | 0.01±0.00 | 0.01±0.00 |
| 9 | Octanal | 0.7 | Fat, green, nut | 18.70±5.63 | 7.97±1.66 | 5.84±0.53 | 3.97±2.91 | 9.86±1.83 | 7.49±0.81 | 7.23±2.18 | 13.37±1.40 | 11.24±1.39 | 15.96±1.64 |
| 10 | (*E*, *E*)-2,4-Heptadienal | 60 | green fruity | 1.71±0.25 | 2.54±0.32 | 2.10±0.07 | 1.89±0.28 | 2.53±0.36 | 2.26±0.20 | 2.33±0.22 | 2.30±0.09 | 2.10±0.45 | 2.31±0.22 |
| 11 | 5-Ethylcyclopent-1-enecarboxaldehyde | / | / | | | | | | | | | | |
| 12 | Benzeneacetaldehyde | 4 | Fruity, floral, honey | | | | | 75.32±11.86 | 111.31±6.13 | 164.66±16.05 | 199.74±4.37 | 165.54±9.20 | 234.36±17.79 |
| 13 | 2,6-Dimethyl-5-heptenal | 16 | Citrus, fruity, green | 0.03±0.00 | 0.02±0.00 | 0.02±0.00 | 0.01±0.00 | 0.03±0.00 | 0.02±0.00 | 0.03±0.00 | 0.02±0.00 | 0.02±0.00 | 0.03±0.00 |
| 14 | (*E*)-2-Octenal | / | Fat, green | | | | | | | | | | |
| 15 | Nonanal | 1 | Floral, fruity | 85.35±4.74 | 70.99±7.97 | 49.57±4.01 | 44.40±8.82 | 87.47±10.88 | 71.32±2.28 | 90.84±10.44 | 179.68±10.53 | 91.41±8.39 | 103.72±2.97 |
| 16 | (2*E*, 6*Z*)-Nona-2,6-dienal | 0.0045 | Fresh, cucumber | | | | 57.59±1.64 | 3774.45±508.59 | 3785.80±245.93 | 4635.61±811.72 | 6084.96±160.27 | 5768.37±334.72 | 7239.38±461.05 |
| 17 | (2*E*)-Non-2-ena | 0.08 | Fresh, cucumber- | | | | 6.22±1.47 | 78.3±11.54 | 76.07±3.20 | 166.46±27.93 | 190.09±10.98 | 181.79±4.38 | 202.17±7.78 |

| | | | | | | | | | | | | | |
|---|---|---|---|---|---|---|---|---|---|---|---|---|---|
| | | | like | | | | | | | | | | |
| 18 | 2,4-Dimethyl-benzaldehyde | / | / | | | | | | | | | | |
| 19 | Decanal | / | / | | | | | | | | | | |
| 20 | 2,4-Nonadienal | 0.1 | Fat, geranium, green | 8.78±2.27 | 12.39±1.65 | 18.66±0.87 | 24.08±2.78 | 17.31±1.57 | 21.72±1.62 | 21.00±4.66 | 22.92±1.58 | 26.78±3.32 | 38.66±1.62 |
| 21 | *β*-Cyclocitral | 5 | Fresh, mint | 0.29±0.03 | 0.39±0.03 | 0.32±0.04 | 0.26±0.03 | 0.29±0.02 | 0.27±0.02 | 0.32±0.04 | 0.36±0.02 | 0.39±0.07 | 0.54±0.06 |
| 22 | Neral | 100 | Citrus, fruit, lemon | 0.24±0.03 | 0.22±0.01 | 0.18±0.01 | 0.28±0.03 | 1.35±0.12 | 1.43±0.09 | 1.63±0.19 | 2.11±0.08 | 1.90±0.05 | 1.36±0.07 |
| 23 | 4-(1-Methylethyl)-benzaldehyde | / | / | | | | | | | | | | |
| 24 | *β*-Homocyclocitral | / | / | | | | | | | | | | |
| 25 | (*E*)-2-Decenal | / | Fat, fish | | | | | | | | | | |
| 26 | Citral | 1 | Fruity, like lemon | 10.14±1.48 | 8.91±0.83 | 6.82±0.74 | 12.69±1.62 | 75.27±6.98 | 79.51±5.42 | 91.21±11.00 | 119.39±4.76 | 107.18±3.14 | 75.74±4.35 |
| 27 | *α*-Ethylidene-benzeneacetaldehyde | / | Cocoa, honey | | | | | | | | | | |
| 28 | Perillaldehyde | 56 | Fat, green, pungent | 0.01±0.00 | 0.00±0.00 | 0.00±0.00 | 0.01±0.00 | 0.03±0.00 | 0.03±0.00 | 0.03±0.00 | 0.03±0.00 | 0.03±0.00 | 0.02±0.00 |
| 29 | Phellandral | / | / | | | | | | | | | | |
| 30 | *α*-Terpinen-7-al | / | Fat, spice | | | | | | | | | | |
| 31 | (2*E*,4*Z*)-Deca-2,4-dienal | 0.04 | Fat, flower, fried | 1.42±0.24 | 3.76±0.72 | 2.45±0.26 | 3.75±0.43 | 5.99±0.59 | 5.23±0.35 | 5.78±0.73 | 6.37±0.50 | 3.96±0.46 | 3.84±0.63 |
| 32 | Undecanal | / | Citrus, pungent | | | | | | | | | | |
| 33 | 2,4-Decadienal | 0.3 | Fat, sweet | 0.37±0.03 | 0.9±0.11 | 0.84±0.08 | 1.36±0.16 | 1.34±0.17 | 1.04±0.10 | 0.98±0.05 | 1.03±0.05 | 0.82±0.08 | 1.47±0.08 |
| 34 | 2-Undecenal | / | Sweet, talc powder | | | | | | | | | | |
| 35 | 2-Butyl-2-octenal | / | Meat | | | | | | | | | | |
| 36 | Dodecanal | 14 | Citrus, fat | 0.02±0.00 | 0.01±0.00 | 0.01±0.00 | 0.01±0.00 | 0.02±0.00 | 0.02±0.00 | 0.02±0.00 | 0.02±0.01 | 0.02±0.00 | 0.02±0.00 |
| 37 | (*Z*)-2-Penten-1-ol | 720 | Banana, green | 0.00±0.00 | 0.00±0.00 | 0.00±0.00 | 0.00±0.00 | 0.00±0.00 | 0.00±0.00 | 0.00±0.00 | 0.00±0.00 | 0.00±0.00 | 0.00±0.00 |
| 38 | (*Z*)-3-Hexen-1-ol | 70 | Grass, green, herb | 6.29±1.07 | 12.87±1.33 | 11.77±0.6 | 13.42±1.46 | 25.85±2.46 | 22.53±0.98 | 18.79±1.29 | 16.98±0.29 | 11.56±0.49 | 5.68±0.29 |

| | | | | | | | | | | | | | |
|---|---|---|---|---|---|---|---|---|---|---|---|---|---|
| 39 | (*E*)-2-Hexen-1-ol | 100 | Fat, fruity, geranium | 0.31±0.05 | 1.49±0.16 | 1.16±0.09 | 2.58±0.27 | 12.14±1.25 | 11.48±0.36 | 10.8±0.97 | 10.73±0.35 | 9.68±0.85 | 3.86±0.25 |
| 40 | 1-Hexanol | / | Fat, flower, fruity | | | | | | | | | | |
| 41 | 2-Heptanol | 70 | Coconut, fried | 1.23±0.34 | 1.24±0.10 | 1.16±0.19 | 1.93±0.36 | 3.53±0.13 | 3.55±0.26 | 3.40±0.42 | 2.69±0.17 | 1.81±0.12 | 1.08±0.03 |
| 42 | 1-Heptanol | 5.6 | Green, putrid, wood | 0.37±0.05 | 1.56±0.22 | 0.93±0.40 | 0.11±0.06 | 0.52±0.11 | 0.54±0.17 | 0.44±0.07 | 0.51±0.05 | 0.26±0.09 | 0.07±0.03 |
| 43 | 1-Octen-3-ol | 1 | Earth, mushroom, | 49.97±2.94 | 36.18±5.45 | 32.66±2.17 | 34.11±4.64 | 79.19±7.10 | 72.43±3.14 | 64.59±5.69 | 75.76±10.69 | 56.89±6.08 | 61.39±6.11 |
| 44 | Benzyl alcohol | 10000 | Fruity | 0.14±0.01 | 0.13±0.01 | 0.11±0.00 | 0.07±0.01 | 0.09±0.01 | 0.06±0.00 | 0.07±0.01 | 0.07±0.02 | 0.07±0.00 | 0.06±0.00 |
| 45 | $\alpha$-Methyl-Benzenemethanol | 700 | Floral, honey, rose | 0.00±0.00 | 0.00±0.00 | 0.00±0.00 | 0.00±0.00 | 0.00±0.00 | 0.00±0.00 | 0.00±0.00 | 0.00±0.00 | 0.00±0.00 | 0.00±0.00 |
| 46 | (*Z*)-Thujane-4-ol | / | Balsamic | | | | | | | | | | |
| 47 | 1-Octanol | 100 | Citrus, fat, fruit | | | | | | | 0.01±0.00 | 0.01±0.00 | 0.01±0.00 | 0.01±0.00 |
| 48 | Linalool oxide Ⅰ | 320 | Sweet | 0.03±0.00 | 0.03±0.00 | 0.03±0.00 | 0.03±0.00 | 0.06±0.01 | 0.05±0.00 | 0.05±0.01 | 0.05±0.00 | 0.05±0.00 | 0.05±0.00 |
| 49 | Linalool oxide II | 320 | Citrus, sweet | 0.06±0.01 | 0.05±0.01 | 0.05±0.00 | 0.05±0.01 | 0.08±0.01 | 0.07±0.00 | 0.08±0.01 | 0.08±0.00 | 0.07±0.00 | 0.07±0.00 |
| 50 | Linalool | 6 | Floral, fruity | 243.34±21.25 | 203.48±27.22 | 165.88±8.01 | 157.93±18.15 | 244.32±21.88 | 209.66±11.10 | 197.97±17.48 | 205.16±3.34 | 168.53±7.17 | 161.75±11.95 |
| 51 | Hotrienol | 110 | Floral, woody | 0.03±0.00 | 0.04±0.01 | 0.02±0.00 | 0.03±0.00 | 0.05±0.01 | 0.04±0.00 | 0.04±0.01 | 0.04±0.00 | 0.04±0.00 | 0.10±0.01 |
| 52 | 2, 6-Dimethyl-cyclohexanol | / | / | | | | | | | | | | |
| 53 | Phenylethyl Alcohol | 1000 | Floral, fruit, honey | 1.64±0.25 | 1.48±0.11 | 1.26±0.10 | 1.06±0.11 | 1.68±0.14 | 1.38±0.07 | 1.49±0.16 | 1.70±0.05 | 1.53±0.06 | 1.52±0.07 |
| 54 | (*Z*)-p-Mentha-2,8-dien-1-ol | / | Fresh, mint | | | | | | | | | | |
| 55 | p-Menth-2-en-1-ol | / | Herb, spice | | | | | | | | | | |
| 56 | (*E*)-Pinocarveol | / | / | | | | | | | | | | |
| 57 | (*Z*)-3-Nonen-1-ol | / | Fresh, green | | | | | | | | | | |
| 58 | Nerol oxide | / | Floral, oil | | | | | | | | | | |
| 59 | 1-Nonanol | 1000 | Fat, floral, green | 0.00±0.00 | 0.00±0.00 | 0.00±0.00 | 0.00±0.00 | 0.00±0.00 | 0.00±0.00 | 0.00±0.00 | 0.00±0.00 | 0.00±0.00 | 0.00±0.00 |
| 60 | Linalool oxide III | / | Citrus, floral, sweet | | | | | | | | | | |

| | | | | | | | | | | | | | |
|---|---|---|---|---|---|---|---|---|---|---|---|---|---|
| 61 | Linalool oxide Ⅳ | / | Floral | | | | | | | | | | |
| 62 | Terpinen-4-ol | 590 | Citrus, floral, herb | 0.22±0.02 | 0.17±0.02 | 0.14±0.01 | 0.14±0.02 | 0.30±0.03 | 0.27±0.02 | 0.32±0.04 | 0.33±0.05 | 0.33±0.01 | 0.35±0.03 |
| 63 | *p*-Cymen-8-ol | / | Citrus, sweet | | | | | | | | | | |
| 64 | *α*-Terpineol | / | Citrus, floral, fresh | | | | | | | | | | |
| 65 | (*Z*)-Piperitol | / | / | | | | | | | | | | |
| 66 | Myrtenol | / | / | | | | | | | | | | |
| 67 | (*E*)-Isopiperitenol | / | / | | | | | | | | | | |
| 68 | (*E*)-Piperitol | / | Herb | | | | | | | | | | |
| 69 | (Z)-Isopiperitenol | / | / | | | | | | | | | | |
| 70 | Nerol | 300 | / | 0.10±0.01 | 0.11±0.02 | 0.07±0.01 | 0.07±0.01 | 0.29±0.01 | 0.30±0.03 | 0.34±0.06 | 0.36±0.02 | 0.32±0.01 | 0.25±0.03 |
| 71 | Citronellol | / | Citrus, floral, green | | | | | | | | | | |
| 72 | (*Z*)-*p*-1(6),8-menthadien-2-ol | 250 | Caraway, cool | | | | | | 0.00±0.00 | 0.00±0.00 | 0.00±0.00 | 0.00±0.00 | 0.00±0.00 |
| 73 | Isogeraniol | / | Rose | | | | | | | | | | |
| 74 | Geraniol | 7.5 | Floral, lemon peel | 46.33±1.71 | 53.14±3.20 | 39.83±3.07 | 43.47±4.65 | 96.75±10.34 | 91.18±6.17 | 93.56±8.37 | 96.21±16.70 | 91.99±1.02 | 95.09±9.20 |
| 75 | *p*-Mentha-1,8-dien-7-ol | / | Fat, green, sweet | | | | | | | | | | |
| 76 | *p*-Menth-1-en-9-ol | / | Fruit, herb, sweet | | | | | | | | | | |
| 77 | Nerolidol | 250 | Floral, hay, wood | 0.03±0.00 | 0.01±0.00 | 0.00±0.00 | 0.00±0.00 | 0.01±0.00 | 0.01±0.00 | 0.01±0.00 | 0.01±0.00 | 0.01±0.00 | 0.01±0.00 |
| 78 | δ-Cadinol | / | Herb, spice | | | | | | | | | | |
| 79 | Methyl-hex-2-enoate | / | Fruity | | | | | | | | | | |
| 80 | Ethyl caproate | / | Fruity | | | | | | | | | | |
| 81 | (*E*)-3-Hexenyl acetate | 870 | Fruity, green | 0.02±0.00 | 0.02±0.00 | 0.01±0.00 | 0.01±0.00 | 0.01±0.00 | 0.00±0.00 | 0.00±0.00 | 0.00±0.00 | | |
| 82 | Hexyl ethanoate | / | Sweet, fruity | | | | | | | | | | |
| 83 | (*E*)-2-Hexenyl acetate | / | / | | | | | | | | | | |
| 84 | Methylbenzoate | 73 | Herb, sweet, violet | 0.00±0.00 | 0.01±0.00 | 0.01±0.00 | 0.00±0.00 | 0.01±0.00 | 0.01±0.00 | 0.01±0.00 | 0.00±0.00 | 0.01±0.00 | 0.00±0.00 |
| 85 | Benzyl acetate | 30 | Floral, fresh, fruit | 0.04±0.00 | 0.03±0.01 | 0.03±0.01 | 0.01±0.00 | 0.05±0.02 | 0.08±0.02 | 0.06±0.03 | 0.09±0.01 | 0.14±0.02 | 0.04±0.01 |

| | | | | | | | | | | | | | |
|---|---|---|---|---|---|---|---|---|---|---|---|---|---|
| 86 | (*E*)-Hex-3-enyl butyrate | / | / | | | | | | | | | | |
| 87 | Hexyl butanoate | 203 | Citrus, fresh | | | 0.01±0.00 | 0.01±0.00 | 0.04±0.00 | 0.03±0.00 | 0.03±0.00 | 0.02±0.00 | 0.01±0.00 | 0.01±0.00 |
| 88 | (*E*)-2-Hexenyl butyrate | / | / | | | | | | | | | | |
| 89 | Methyl salicylate | 40 | Fresh, mint | 7.07±0.64 | 8.04±0.60 | 7.77±0.56 | 7.75±0.85 | 17.27±1.30 | 15.9±0.75 | 15.98±1.72 | 16.17±0.39 | 14.33±0.52 | 12.97±0.67 |
| 90 | Ethyl nonanoate | 70 | Apple, apricot, fresh | 0.02±0.00 | 0.01±0.00 | 0.01±0.00 | 0.01±0.00 | 0.00±0.00 | 0.01±0.00 | 0.00±0.00 | 0.02±0.02 | 0.00±0.00 | 0.01±0.00 |
| 91 | (*Z*)-3-Hexenyl-*α*-methylbutyrate | / | Herb, sweet | | | | | | | | | | |
| 92 | (*E*)-2-Hexenyl isovalerate | / | / | | | | | | | | | | |
| 93 | Hexyl 2-methylbutanoate | 22 | / | 0.00±0.00 | 0.01±0.00 | 0.01±0.00 | 0.01±0.00 | 0.02±0.01 | 0.04±0.00 | 0.03±0.02 | 0.04±0.00 | 0.03±0.00 | 0.03±0.00 |
| 94 | Phenethyl acetate | 249.6 | Floral, fruit, rose | 0.00±0.00 | 0.00±0.00 | 0.00±0.00 | 0.00±0.00 | 0.00±0.00 | 0.00±0.00 | 0.00±0.00 | 0.00±0.00 | 0.00±0.00 | 0.00±0.00 |
| 95 | Ethyl salicylate | 84 | Fruity, mint | 0.00±0.00 | 0.00±0.00 | 0.00±0.00 | 0.00±0.00 | 0.01±0.00 | 0.01±0.00 | 0.01±0.00 | 0.01±0.00 | 0.01±0.00 | 0.00±0.00 |
| 96 | Prenyl hexanoate | / | / | | | | | | | | | | |
| 97 | Ethyl pelargonate | 377 | Banana, fruit | 0.00±0.00 | 0.00±0.00 | 0.00±0.00 | 0.00±0.00 | 0.00±0.00 | 0.00±0.00 | 0.00±0.00 | 0.00±0.00 | 0.00±0.00 | 0.00±0.00 |
| 98 | Methyl geranate | 21.44 | Fruity, fresh | 2.49±0.21 | 1.41±0.08 | 1.05±0.09 | 1.67±0.22 | 5.24±0.49 | 5.94±0.12 | 6.89±0.68 | 8.09±0.32 | 7.56±0.31 | 6.96±0.58 |
| 99 | 3-Hydroxy-2,2,4-trimethylpentyl isobutyrate | / | / | | | | | | | | | | |
| 100 | (Z)-3-Hexenyl hexanoate | / | Fruity, prune | | | | | | | | | | |
| 101 | Hexanoic acid, hexyl ester | / | Apple peel, peach | | | | | | | | | | |
| 102 | (Z)-3-Hexenyl (Z)-3-hexenoate | / | / | | | | | | | | | | |
| 103 | (*E*)-2-Hexenyl Hexanoate | / | / | | | | | | | | | | |
| 104 | β-Phenylethyl butyrate | / | Fruity | | | | | | | | | | |
| 105 | Dimethyl phthalate | / | / | | | | | | | | | | |
| 106 | (Z)-3-Hexenyl benzoate | / | Green, spice | | | | | | | | | | |
| 107 | Benzoic acid, hexyl ester | / | Green wood, pine | | | | | | | | | | |
| 108 | (*E*)-2-Hexenyl benzoate | / | / | | | | | | | | | | |

| | | | | | | | | | | | | | |
|---|---|---|---|---|---|---|---|---|---|---|---|---|---|
| 109 | 2-Heptanone | / | Bell pepper, green | | | | | | | | | | |
| 110 | 6-Methyl-5-hepten-2-one | 100 | Mushroom, pepper | | | | | | | | 0.01±0.00 | 0.02±0.01 | 0.01±0.00 |
| 111 | 3-Octanone | 28 | Butter, herb, sweet | 0.00±0.00 | 0.00±0.00 | 0.00±0.00 | 0.00±0.00 | 0.00±0.00 | 0.00±0.00 | 0.00±0.00 | 0.00±0.00 | 0.00±0.00 | 0.00±0.00 |
| 112 | 3-Octen-2-one | / | Green, nut, rose | | | | | | | | | | |
| 113 | 3-Methyl-2-cyclohexen-1-one | / | / | | | | | | | | | | |
| 114 | Acetophenone | 65 | Floral, must, plastic | 0.00±0.00 | 0.01±0.00 | 0.01±0.00 | 0.00±0.00 | 0.02±0.00 | 0.01±0.00 | 0.01±0.00 | 0.01±0.00 | 0.01±0.00 | 0.01±0.00 |
| 115 | (*E*, *E*)-3,5-Octadien-2-one | 100 | Balsamic, dill, sweet | 0.05±0.00 | 0.05±0.01 | 0.05±0.00 | 0.04±0.00 | 0.03±0.00 | 0.03±0.00 | 0.02±0.00 | 0.02±0.00 | 0.02±0.00 | 0.03±0.00 |
| 116 | 3,5-Octadien-2-one | / | Fat, mushroom | | | | | | | | | | |
| 117 | 5-Ethyl-6-methyl-3E-hepten-2-one | / | Citrus | | | | | | | | | | |
| 118 | 5-Isopropylbicyclo [3.1.0]hexan-2-one | / | / | | | | | | | | | | |
| 119 | Carvone | 1 | Caraway | 0.05±0.00 | 0.05±0.01 | 0.04±0.01 | 0.07±0.01 | 0.42±0.04 | 0.37±0.07 | 0.35±0.10 | 0.34±0.06 | 0.34±0.08 | 0.32±0.03 |
| 120 | Isopiperitenone | / | / | | | | | | | | | | |
| 121 | 2-Undecanone | / | Fresh, green | | | | | | | | | | |
| 122 | *β*-Damascenone | 0.002 | Floral, fruit, honey | | | | | | | | 697.39±162.84 | | 343.21±66.5 |
| 123 | (*Z*)-Jasmone | 1.9 | Floral, like jasmine | 160.88±12.61 | 95.25±12.21 | 70.53±3.15 | 65.44±7.34 | 118.34±10.18 | 93.71±5.86 | 98.86±14.58 | 106.27±3.76 | 90.77±4.48 | 89.49±7.53 |
| 124 | *α*-Ionone | 0.4 | Floral, fruit | 7.58±0.70 | 10.03±0.77 | 8.37±1.14 | 7.77±0.87 | 7.07±0.57 | 6.18±0.33 | 7.15±1.24 | 8.92±0.70 | 7.72±0.80 | 7.75±0.76 |
| 125 | Geranylacetone | 60 | Green, magnolia | 0.01±0.00 | 0.02±0.00 | 0.01±0.00 | 0.01±0.00 | 0.02±0.00 | 0.02±0.00 | 0.02±0.00 | 0.02±0.00 | 0.02±0.00 | 0.02±0.00 |
| 126 | (*E*)-*β*-Ionone | 0.007 | / | 2938.01±174.56 | 4520.28±435.7 | 3952.33±590.46 | 3225.32±354.67 | 3272.72±323.27 | 3043.48±164.9 | 3809.23±691.13 | 4763.08±219.17 | 4289.59±344.18 | 4453.36±519.56 |
| 127 | 1,2,5,5-Tetramethyl-1,3-cyclopentadiene | / | / | | | | | | | | | | |

| | | | | | | | | | | | | | |
|---|---|---|---|---|---|---|---|---|---|---|---|---|---|
| 128 | Styrene | / | Balsamic, gasoline | | | | | | | | | | |
| 129 | *α*-Thujene | / | Green, herb, wood | | | | | | | | | | |
| 130 | *α*-Pinene | / | Cedarwood, pine | | | | | | | | | | |
| 131 | Dehydrosabinene | / | / | | | | | | | | | | |
| 132 | Sabinene | 75 | Citrus, green, herb | 0.01±0.00 | 0±0.00 | 0.00±0.00 | 0.00±0.00 | 0.00±0.00 | 0.00±0.00 | 0.00±0.00 | 0.00±0.00 | 0.00±0.00 | 0.00±0.00 |
| 133 | *β*-Myrcene | 42 | Balsamic, geranium | 0.01±0.00 | 0.00±0.00 | 0.00±0.00 | 0.00±0.00 | 0.01±0.00 | 0.01±0.00 | 0.01±0.00 | 0.02±0.00 | 0.01±0.00 | 0.01±0.00 |
| 134 | *α*-Phellandrene | 40 | Citrus, fresh, mint | 0.02±0.00 | 0.02±0.00 | 0.01±0.00 | 0.01±0.00 | 0.04±0.01 | 0.02±0.00 | 0.03±0.00 | 0.02±0.00 | 0.02±0.00 | 0.02±0.00 |
| 135 | *α*-Terpinene | 80 | Berry, lemon, wood | 0.10±0.01 | 0.08±0.01 | 0.06±0.00 | 0.06±0.01 | 0.11±0.01 | 0.09±0.00 | 0.10±0.01 | 0.10±0.00 | 0.09±0.01 | 0.10±0.01 |
| 136 | D-Limonene | / | Citrus, mint | | | | | | | | | | |
| 137 | *β*-Phellandrene | 36 | Mint, terpentine | 0.03±0.00 | 0.02±0.00 | 0.02±0.00 | 0.03±0.02 | 0.03±0.01 | 0.04±0.01 | 0.03±0.00 | 0.08±0.06 | 0.03±0.00 | 0.04±0.00 |
| 138 | *β*-Ocimene | / | Herb, sweet | | | | | | | | | | |
| 139 | (*E*)-β-Ocimene | 34 | Citrus, herb, sweet | 0.03±0.00 | 0.02±0.00 | 0.01±0.00 | 0.02±0.00 | 0.03±0.00 | 0.03±0.00 | 0.03±0.00 | 0.03±0.00 | 0.02±0.00 | 0.03±0.00 |
| 140 | γ-Terpinene | 1000 | Bitter, citrus | 0.01±0.00 | 0.01±0.00 | 0.01±0.00 | 0.01±0.00 | 0.01±0.00 | 0.01±0.00 | 0.01±0.00 | 0.01±0.00 | 0.01±0.00 | 0.01±0.00 |
| 141 | Isoterpinene | 200 | Pine, plastic, sweet | 0.01±0.00 | 0.01±0.00 | 0.01±0.00 | 0.01±0.00 | 0.01±0.00 | 0.01±0.00 | 0.01±0.00 | 0.01±0.00 | 0.01±0.00 | 0.01±0.00 |
| 142 | 1,3,8-p-Menthatriene | 15 | Turpentine | 0.05±0.01 | 0.05±0.00 | 0.03±0.00 | 0.03±0.00 | 0.04±0.00 | 0.04±0.00 | 0.04±0.00 | 0.04±0.00 | 0.04±0.00 | 0.06±0.00 |
| 143 | (*E*)-4,8-Dimethylnona-1,3,7-triene | / | / | | | | | | | | | | |
| 144 | (*E*, *E*)-2,6-Dimethyl-1,3,5,7-octatetraene | / | / | | | | | | | | | | |
| 145 | Safrole | / | Spice, sweet, warm | | | | | | | | | | |
| 146 | 1-Ethylidene-1H-indene | / | / | | | | | | | | | | |
| 147 | *α*-Cubebene | / | Herb, wax, wood | | | | | | | | | | |
| 148 | 1-Tetradecene | 60 | / | 0.00±0.00 | 0.00±0.00 | 0.00±0.00 | 0.00±0.00 | 0.00±0.00 | 0.00±0.00 | 0.00±0.00 | 0.00±0.00 | 0.00±0.00 | 0.00±0.00 |
| 149 | Caryophyllene | 64 | Fried, spice, wood | 0.00±0.00 | 0.00±0.00 | 0.00±0.00 | 0.00±0.00 | 0.00±0.00 | 0.00±0.00 | 0.00±0.00 | 0.00±0.00 | 0.00±0.00 | 0.00±0.00 |

| | | | | | | | | | | | | | |
|---|---|---|---|---|---|---|---|---|---|---|---|---|---|
| 150 | Longifolene | / | Floral, vegetable | | | | | | | | | | |
| 151 | Cedrene | / | / | | | | | | | | | | |
| 152 | (*E*)-*β*-Farnesene | / | Citrus, floral, fresh | | | | | | | | | | |
| 153 | *α*-Muurolene | / | / | | | | | | | | | | |
| 154 | *α*-Farnesene | / | Citrus, green, herb | | | | | | | | | | |
| 155 | (*E*)-Calamenene | / | / | | | | | | | | | | |
| 156 | δ-Cadinene | / | Wood | | | | | | | | | | |
| 157 | *α*-Calacorene | / | Wood | | | | | | | | | | |
| 158 | Toluene | 6 | Glue, paint | 0.04±0.01 | 0.02±0.00 | 0.02±0.00 | | | | | | | |
| 159 | *p*-Xylene | / | Metal, sweet | | | | | | | | | | |
| 160 | *p*-Cymene | / | Citrus, fresh, wood | | | | | | | | | | |
| 161 | 1-Methyl-4-(1-methylethenyl)-benzene | 85 | Citrus, petrol, pine | 0.02±0.00 | 0.02±0.00 | 0.02±0.00 | 0.02±0.00 | 0.03±0.00 | 0.03±0.00 | 0.03±0.00 | 0.03±0.00 | 0.03±0.00 | 0.05±0.01 |
| 162 | Naphthalene | 50 | Pungent | 0.03±0.00 | 0.03±0.00 | 0.02±0.00 | 0.01±0.00 | 0.02±0.00 | 0.02±0.00 | 0.01±0.00 | 0.02±0.00 | 0.01±0.00 | 0.02±0.00 |
| 163 | 2-Methyl-naphthalene | 3 | Pungent, root | 0.26±0.03 | 0.27±0.03 | 0.20±0.02 | 0.10±0.01 | 0.17±0.03 | 0.14±0.02 | 0.12±0.01 | 0.14±0.02 | 0.12±0.00 | 0.08±0.02 |
| 164 | Biphenyl | 0.5 | / | 0.37±0.07 | 0.37±0.03 | 0.25±0.02 | 0.14±0.01 | 0.21±0.04 | 0.17±0.03 | 0.14±0.01 | 0.18±0.00 | 0.15±0.01 | 0.11±0.01 |
| 165 | Fluorene | / | / | | | | | | | | | | |
| 166 | Nonanoic acid | 3000 | Fat, green，sweat | | | | | 0.00±0.00 | 0.00±0.00 | 0.00±0.00 | 0.00±0.00 | 0.00±0.00 | 0.00±0.00 |
| 167 | Geranic acid | / | / | | | | | | | | | | |
| 168 | Octane | / | Fat, oil, sweet | | | | | | | | | | |
| 169 | 2,2,4,6,6-Pentamethyl-heptane | / | / | | | | | | | | | | |
| 170 | Dodecane | / | Alkane, undesirable | | | | | | | | | | |
| 171 | 2,6,11-trimethyl-Dodecane | / | / | | | | | | | | | | |
| 172 | Tridecane | / | Alkane | | | | | | | | | | |
| 173 | 3-Methyl-tridecane | / | / | | | | | | | | | | |

| | | | | | | | | | | | | | |
|---|---|---|---|---|---|---|---|---|---|---|---|---|---|
| 174 | Tetradecane | / | / | | | | | | | | | | |
| 175 | Octylcyclohexane | / | / | | | | | | | | | | |
| 176 | 3-Methyl-pentadecane | / | / | | | | | | | | | | |
| 177 | Hexadecane | / | Alkane, root | | | | | | | | | | |
| 178 | Heptadecane | / | Alkane, pungent | | | | | | | | | | |
| 179 | 2-n-Butyl furan | 5 | Wet hay | 0.07±0.01 | 0.05±0.00 | 0.06±0.02 | 0.05±0.01 | 0.03±0.01 | 0.02±0.00 | 0.02±0.00 | 0.04±0.01 | | |
| 180 | 2-Pentyl-furan | 6 | Floral, fruit, green | 0.29±0.00 | 0.29±0.05 | 0.33±0.03 | 0.39±0.05 | 0.32±0.04 | 0.27±0.05 | 0.27±0.06 | 0.28±0.02 | 0.35±0.03 | 0.58±0.05 |
| 181 | 1,8-Cineole | / | Fresh, mint | | | | | | | | | | |
| 182 | 2,3-Dihydro-benzofuran | / | / | | | | | | | | | | |
| 183 | Dibenzofuran | 3.3 | Rotten, rubber | 0.03±0.00 | 0.02±0.00 | 0.02±0.00 | | | | | | | |
| 184 | Camphor | / | / | | | | | | | | | | |
| 185 | Benzothiazole | 80 | Nut, rubber | 0.00±0.00 | 0.00±0.00 | 0.00±0.00 | 0.00±0.00 | 0.00±0.00 | 0.00±0.00 | 0.00±0.00 | 0.00±0.00 | 0.00±0.00 | 0.00±0.00 |
| 186 | Indole | / | Fecal, floral | | | | | | | | | | |
| 187 | Dihydro-5-pentyl-2(3H)-furanone | 25 | Peach, sweet | | | 0.00±0.00 | 0.00±0.00 | 0.01±0.00 | 0.01±0.00 | 0.01±0.00 | 0.01±0.00 | 0.01±0.00 | 0.01±0.00 |
| 188 | 2,4-Di-tert-butylphenol | / | / | | | | | | | | | | |
| 189 | 5-Ethyldihydro-2(3h)-furanone | 1600 | Onion, sweet | | | 0.00±0.00 | 0.00±0.00 | 0.00±0.00 | 0.00±0.00 | 0.00±0.00 | 0.00±0.00 | 0.00±0.00 | 0.00±0.00 |

Thresholds: Odor thresholds in water. The values were obtained from published literature or relevant websites.
